# Natural deep eutectic solvent supported targeted solid–liquid polymer carrier for breast cancer therapy†

**DOI:** 10.1039/d0ra03790g

**Published:** 2020-10-07

**Authors:** Xianfu Sun, Periyakaruppan Pradeepkumar, Naresh Kumar Rajendran, Harshavardhan Shakila, Nicolette Nadene Houreld, Dunia A. Al Farraj, Yousif M. Elnahas, Nandhakumar Elumalai, Mariappan Rajan

**Affiliations:** Department of Breast, The Affiliated Tumor Hospital of Zhengzhou University Zhengzhou Henan 450008 China; Biomaterials in Medicinal Chemistry Laboratory, Department of Natural Products Chemistry, School of Chemistry, Madurai Kamaraj University Madurai-625021 Tamil Nadu India rajanm153@gmail.com; Laser Research Centre, Faculty of Health Sciences, University of Johannesburg PO Box 17011 Doornfontein 2028 South Africa; Department of Molecular Microbiology, School of Biotechnology, Madurai Kamaraj University Madurai-625021 India; Department of Botany and Microbiology, College of Science, King Saud University Riyadh 11451 Saudi Arabia; Department of Surgery, College of Medicine, King Saud University Medical City, King Saud University Riyadh Saudi Arabia; Department of Biochemistry, Sri Muthukumaran Medical College and Research Institute Chennai-600069 Tamil Nadu India

## Abstract

Solid–liquid nanocarriers (SLNs) are at the front of the rapidly emerging field of medicinal applications with a potential role in the delivery of bioactive agents. Here, we report a new SLN of natural deep eutectic solvent (NADES) and biotin-conjugated lysine–polyethylene glycol copolymer. The SLN system was analyzed for its functional groups, thermal stability, crystalline nature, particle size, and surface morphology through the instrumental analysis of FT-IR, TGA, XRD, DLS, SEM, and TEM. Encapsulation of PTX (paclitaxel) and 7-HC (7-hydroxycoumarin) with the SLN was carried out by dialysis, and UV-visible spectra evidenced the drug loading capacity and higher encapsulation efficiency obtained. The enhanced anticancer potential of PTX- and 7-HC-loaded SLN was assessed *in vitro*, and the system reduces the cell viability of MDA-MB-231 cells. The PTX- and 7-HC-loaded SLN system was investigated in a breast cancer-induced rat model *via in vivo* studies. It shows decreased lysosomal enzymes and increased levels of caspase to cure breast tumors. It very well may be reasoned that the designed PTX- and 7-HC-loaded SLN system has strong anticancer properties and exhibits potential for delivery of drug molecules in cancer treatment.

## Abstract 2

Solid–liquid nanocarriers (SLNs) are at the front of the rapidly emerging field of medicinal applications with a potential role in the delivery of bioactive agents.

## Graphical abstract

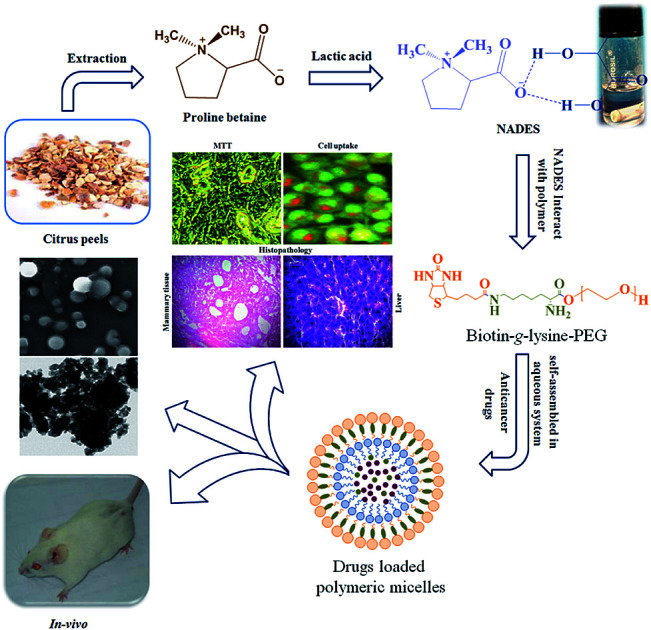

## Introduction

Recent research development in medicinal applications has advanced through the improvement of new methods and materials that are more biodegradable, biocompatible, and less toxic.^1,2^ There is an enormous attempt to explore the role/function of synthetic biomaterials used in drug delivery applications.^3^ Currently, synthetic and natural polymers, metal complexes, peptides, proteins, and hydrogels are used for the development of anticancer drug delivery systems,^4–8^ which reduce some disadvantages over conventional anticancer treatments such as poor solubility, low biodegradability, toxic nature, and poor intracellular penetration, retention time and sustainable release of encapsulated drugs.^9^ A significant number of nanoparticles such as inorganic nanoparticles or other polymeric/liquid nanoparticles have also been used in developing drug carrier systems. The drug carrier leads to the fabrication of a nanocarrier, and most of these are made up of synthetic polymers and not liquid-based carriers.^10^

Researchers have paid more attention to the advancement of new carrier systems by using ILs (ionic liquids) and DESs (deep eutectic solvents).^11^ Unfortunately, ILs and DESs have exhibited thermal instability, low drug loading levels, low drug release and solubility, and have a very weak interaction with and a toxic nature in biological systems. This issue can be overcome by the utilization of natural deep eutectic solvents (NADES). A NADES is a highly biocompatible material designed to serve as a carrier molecule that transports drugs to a specific site without any side effects; it is a non-toxic solvent prepared by secondary metabolites and does not affect the drug release mechanism.^12^ Secondary metabolites like phenolics, terpenoids, flavonoids, and other natural compounds are crucial for medicinal applications.^13,14^ Compared to organic solvents, the use of NADES has gained attention in the synthesis of carrier systems.^15–18^

*Citrus limon* peels are rich in bioactive compounds, including antioxidants, flavonoids, vitamins, and prolinebetaine (PB). PB is a natural bioactive compound, which has numerous biological properties, including antifungal, antibacterial, and anticancer activities.^19,20^ Anticancer activity is an essential property of PB and is due to the presence of amine which is a highly reactive compound that can readily react with biological compound moieties such as hydroxyl groups. In this study, PB is isolated and used for the synthesis of NADES in combination with lactic acid (LA), which is an excellent electron donor/acceptor. The self-assembled drug carrier system with PB is reported to target tumor cells.^21^ However, there is the need for an extra efficient and active targeting ligand to achieve better cellular uptake of drug-loaded carriers. Different ligands such as polysaccharides, folic acid, peptides, and biotin are involved in targeting drug carrier synthesis.^22^ Among these, biotin is predominantly utilized as a tumor-focusing group for numerous cancer chemotherapy applications.^23^ Tumor cells need vitamins for their survival, and speedily growing cancer cells overexpress the receptors for this vitamin on their cell surface.

This paper reports for the first time on the preparation of NADES using secondary metabolites, and it is used as a biotin-conjugated solid–liquid nanocarrier (SLN) for the encapsulation of the anticancer drugs paclitaxel (PTX) and 7-hydroxycoumarin (7-HC). The advantage of SLNs is the ability to incorporate hydrophobic anticancer drugs in the inner core and hydrophilic anticancer drugs on the outer core. The incorporation of PTX and 7-HC in the SLN system will reduce side effects and improve bioavailability. The anticancer activity of the synthesized PTX- and 7-HC-loaded SLN system was investigated *in vitro* in a MDA-MB-231 breast cancer cell line, and *in vivo* in a DMBA-induced breast cancer rat model.

## Experimental section

### Materials

EDC (1-ethyl-3-(3-dimethylaminopropyl)carbodiimide), LA (lactic acid), NHS (*N*-hydroxysuccinimide), *p*-TsOH (*p*-toluenesulfonic acid), tin(ii) 2-ethylhexanoate (Sn(Oct)_2_), and 7,12-dimethylbenz[*a*] anthracene (DMBA) were procured from Sigma-Aldrich, Mumbai, India. Lysine, polyethylene glycol-6000, biotin, PTX, and 7-HC were purchased from Merck, Mumbai, India. Petroleum ether (PE), dichloromethane (DCM), ethyl acetate (EA), diethyl ether (DE), methanol, acetone, acetonitrile (AN) and dimethyl sulfoxide (DMSO) were received from Himedia Laboratories Pvt. Ltd, Mumbai, India (HPLC grade). A human breast adenocarcinoma (MDA-MB-231) cell line and normal mouse fibroblast cell line (L929) obtained from the National Center for Cell Science, Pune, India were used for this work. Caspase colorimetric assay kit was from R and D Systems Inc., USA. The chemicals and solvents used were of analytical grade and main clarity. In all the experiments, double-distilled (DD) water was used as a solvent and washing solution. The other chemicals were purchased from the different companies like BDH Division (Mumbai, India), Glaxo Laboratories, Sisco Research Laboratories, Sarabhai Chemicals (Vadodara, India), and SD Fine Chemicals (Mumbai, India).

### Isolation of prolinebetaine from *Citrus limon* peels

Citrus fruits (*Citrus limon* peels) were acquired from a nearby market at Madurai, Tamil Nadu, India.^24^ The fruits were cleaned with DD water and acetone. The fruit peels were chopped into little pieces and dried at room temperature (27 °C). Dried samples were powdered into fine particles utilizing a blender. Ground samples were then dissolved in various solvents such as petroleum ether, chloroform, ethyl acetate, methanol, acetonitrile, and water for solubility optimization. Samples were loaded onto a silica gel packed column (100–200 mesh). Pet ether was used for initial elution. Fractions were removed for primary compounds such as oils, fats, fatty acids, resin, and chlorophyll compounds. Solvents were recovered through vacuum rotary evaporation (Rotavapor® R-300).

Fractions were collected and used for further analysis. Optimized and stabilized ethyl acetate and methanol in ratios of 70 : 30 and 50 : 50 were used for the collection of 4-hydroxyprolinebetaine and PB. The solvents from the collected samples were evaporated at room temperature (27 °C). The derived compounds were identified by HPLC, ^1^H- and ^13^C-NMR, and FT-IR analysis.

### Synthesis of NADESs

NADESs were prepared according to a previously reported method.^25^ Isolated and purified PB and LA were mixed in molar ratios of 1 : 1, 1 : 2, 1 : 3, and 2 : 1 at 40 °C. Mixtures were magnetically stirred until a transparent homogenous liquid was obtained. Afterward, the eutectic mixture was chilled to room temperature. Ratios of 1 : 2 and 1 : 3 formed a clear solution, while proportions of 1 : 1 and 2 : 1 did not create a clear solution, as is shown in ESI Fig. 1.† The formation of NADESs and the chemical structures were identified through ^1^H and ^13^C NMR and FT-IR spectroscopy, as shown in ESI Fig. 3 and 4.†

### Synthesis of biotin-*g*-lysine

Biotin grafted lysine was prepared by the condensation method and carried out in the presence of EDC·HCl and NHS as per previous literature.^26^ In general, biotin (0.2 g, 0.00136 mmol) and lysine (0.33 g, 0.00136 mmol) were solubilized in DCM (15 mL). To this mixture, EDC·HCl (0.168 mL, 0.00136 mmol) and NHS (0.15 g, 0.00136 mmol) were added drop-wise. The pH of the reaction mixture was adjusted to pH 7.4 using 1 N NaOH and stirred for 2 h at 45 °C. Then, the reaction mixture was precipitated using an organic solvent (acetone). The precipitate was filtered using Whatman (0.2 μm) filter paper and dried in a vacuum chamber. The obtained precipitate was dialyzed using a dialysis membrane (MWCO-12000) in DD water for 22 h to eliminate unreacted constituents.

### Synthesis of biotin-*g*-lysine-*co*-polyethylene glycol (biotin-*g*-lysine-*co*-PEG)

Typically, biotin-*g*-lysine (1 g, 0.00268 mmol), *p*-TsOH (0.4 g, 0.00268 mmol), and polyethylene glycol-6000 (1 g) in 50 mL toluene were placed in a double neck round reaction flask with a Dean–Stark apparatus.^27^ Then, Sn(Oct)_2_ (0.8 mL, 1.0857 mmol) was added drop-wise, and heated at 125 °C for 24 h to reflux the mixture with continuous stirring and elimination of water. The reaction mixture was cooled to room temperature (27 °C); subsequently, the reaction solution was dissolved in ether and filtered to remove by-products. A Soxhlet apparatus was used for polymer extraction for 24 h with an organic solvent (ethyl acetate). Then, the obtained crude product was dried in a vacuum and the chemical structure of biotin-*g*-lysine-*co*-PEG was confirmed through FT-IR spectroscopy.

### NADES-based biotin-conjugated solid–liquid polymer in solvent emulsion method

NADES-based biotin-conjugated polymeric SLN was prepared by the method mentioned in our previous report.^28^ Initially, 1 g of biotin-*g*-lysine-*co*-PEG was dissolved in 1 mL of NADES. This reaction mixture was kept under continuous stirring for 1 h at room temperature until the mixture became a homogeneous solution and attained a viscous consistency. Nanocarriers were formed using dialysis in aqueous solution, following a previous report.^29^ Briefly, 100 mg of NADES-biotin-*g*-lysine-*co*-PEG system was dispersed in 1 mL DMSO solution and the solution was placed into a dialysis bag and dialyzed against 250 mL of DD water for 24 h to eliminate the DMSO solvent. The separated SLN was used for further processing.

### Determination of self-assembly of carrier formation

The critical nanocarrier concentration (CMC) value of NADES-based biotin-conjugated solid–liquid polymer nanocarrier was studied by fluorescence spectroscopy through pyrene as a fluorescent moiety.^30^ In brief, various concentrations of NADES-based biotin-conjugated solid–liquid polymer nanocarrier (0.1 mL to 1.0 mL) were dissolved in 10 mL of DMSO solution. Then, pyrene (0.1 mg) was solubilized in 5 mg mL^−1^ methanol and added to each test container of NADES-based biotin-conjugated solid–liquid polymer nanocarrier solution. The reaction solution was magnetically stirred at 27 °C for 1 day, and solvents vaporized. Afterwards, 5 mL of distilled water was added individually in a sample tube. The fluorescence of every pyrene-containing NADES-based biotin-conjugated solid–liquid polymer nanocarrier solution was determined by emission spectra (spectrofluorometer, Shimadzu F-4500, Japan) at a wavelength of 472 nm at 21 ± 1 °C.

### Encapsulation efficiency (EE)

The NADES-based biotin-conjugated solid–liquid polymer nanocarrier (100 mg) was taken in a 25 mL beaker and PTX (20 mg mL^−1^) and 7-HC (20 mg mL^−1^) dispersed in ethanol were added to the SLN and stirred for 1 h with a magnetic stirrer. For determining the drug encapsulation efficiency, the supernatant solution was collected at 10 min intervals and analyzed by UV-visible spectroscopy at *λ*_max_ values of 270 nm and 320 nm (Shimadzu UV 1800).^16^ EE was calculated with the following equation:^1^1
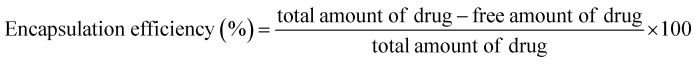


### Drug release study

The discharge of PTX and 7-HC from the nanocarrier was transferred to the dialysis system and a physiological buffer solution at pH values of 2.8, 5.5, and 7.4 was used at 27 °C. Briefly, PTX- and 7-HC-loaded nanocarrier samples (30 mg) were added to 5 mL of fresh PBS (pH 2.8, 5.5, and 7.4) in the dialysis bag at room temperature. The dialysis membrane was incubated in 50 mL PBS. PTX- and 7-HC-loaded SLN samples were stirred using a magnetic stirrer at a constant rate (100 rpm), and 3 mL of the supernatant solution was extracted at standard time intervals and supplemented with 3 mL of new buffer solution. Drugs released into the medium were quantified by a UV-visible spectrometer at *λ*_max_ values of 270 nm and 320 nm (Shimadzu 1600, Japan).

### Swelling studies

The swelling behavior of PTX- and 7-HC-loaded SLN (30 mg) was analyzed in various buffer solutions at pH 2.8, 5.5, and 7.4 at room temperature (27 °C). At standard time intervals, samples were removed and weighed. A stable weight was achieved and the following equation was used to determine the swelling behavior:2
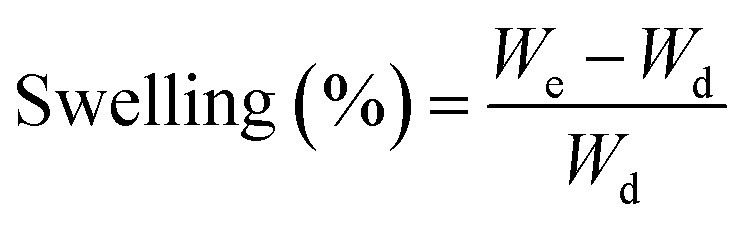
where *W*_e_ and *W*_d_ are the weights of swollen polymer and dried nanocarriers, respectively.

### Biological characterizations

#### Cell culture

A human breast adenocarcinoma cell line (MDA-MB-231) and a mouse fibroblast cell line (L929) were purchased from the NCCS (National Center for Cell Science, Pune, India). Cells were maintained in Dulbecco's modified Eagle's medium supplemented with 10% fetal bovine serum, and 1 unit per mL penicillin and 1 mg mL^−1^ streptomycin, and incubated at 37 °C with 5% CO_2_ in a humidified chamber. Cells were passaged every 3 to 4 days using trypsin/ethylenediaminetetraacetic acid (EDTA) for detachment.

#### Cell viability

MDA-MB-231 human breast adenocarcinoma and L929 normal mouse fibroblast cells were used to interrogate the cytotoxic effect of PTX- and 7-HC-loaded and unloaded SLN system. Cells were seeded into 96-well plates at a seeding density of 1 × 10^5^ cells per well and incubated for 24 h at 37 °C in 5% CO_2_. Various concentrations of PTX- and 7-HC-loaded SLN system (10, 25, 50, 75, and 100 μg mL^−1^) were added to wells and cells were cultured for 24 h. Afterwards, 25 μL of MTT (3-(4,5-dimethylthiazol-2-yl)-2,5-diphenyltetrazolium bromide) assay solution was added to each well and incubated for 5 h at 37 °C. The cell culture medium was measured at an absorption wavelength of 570 nm using a microplate reader (SpectraMax M3, Molecular Devices, USA). The corresponding cell viability (%) was correlated to normal cells. The following equation for cell viability was used for IC_50_ calculation:3
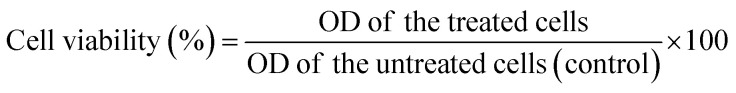


Mean OD of the untreated cells (control), is the mean of the untreated cells used as the control.

#### Cellular uptake analysis by confocal microscope

MDA-MB-231 cells were cultured in 96-well plates and incubated with PTX- and 7-HC-loaded SLN for 24 h. Cells were washed three times with PBS, fixed with 5% paraformaldehyde for 30 min, and stained with 4,6-diamidino-2-phenylindole (DAPI; 1 μg mL^−1^ in PBS) for 10 min. Cells were examined by confocal laser scanning microscopy (Olympus IX 81 under DU897 mode, Japan).

#### Animals and experimental design

Female Sprague-Dawley rats (eight weeks old) were separated into four groups, with each group having six animals. The regimen dose is shown in Table 1. All groups of animals had taken food and water, which was monitored daily for 14 days. A permanent amount of rat chow and fluid was given to every rat and refilled after that day. After treatment periods of 6 weeks, the rats were fasted for two nights, anesthetized and sacrificed by cervical decapitation. Whole blood samples were collected from both separations like plasma/serum, respectively. The mammary tissue was cut and washed in ice-cold saline and weighed. Tissue was cut up and homogenized with 0.1 M Tris–HCl buffer (pH 7.4, and 10%) and centrifuged (3000 × *g* for 10 min). The finally obtained supernatant solution was used for enzyme assays. The bodyweights of the whole animals were recorded before and after the treatment and sacrifice.

**Table 1:** Experimental animal groups and treatment conditions^a^

Group	Group name	Treatment condition
Group I	Control	Intraperitoneal injection of 1 mL saline per week for four weeks
Group II	Induced	Mammary carcinoma induced by a single dose of intraperitoneal injection of 25 mg per kg b.wt of DMBA dissolved in saline
Group III	SLN unloaded	Mammary carcinoma induced same as group II and treated with an intraperitoneal injection of unloaded nanocarriers (5 mg per kg b.wt per week for four-six weeks)
Group IV	SLN loaded	Mammary carcinoma induced same as group II and treated with an intraperitoneal injection of PTX- and 7-HC-loaded SLN (5 mg per kg b.wt per week for six weeks)

a b.wt, body weight; DMBA, 7,12-dimethylbenz[*a*]anthracene.

The experimental work was performed with ethical norms approved by committee members of the Animals (CPCSEA), Ministry of Environment and Forests (Animal Welfare Division), Government of India, and Institutional Animal Ethics Committee (IEAC) Guidelines.

#### Gross observations and tumor volume

The change in the body weight and tumor of animals was measured and recorded in grams. The following formula was used to calculate tumor volume:*v* = 4/3π*r*_12_*r*_2_ (radius *r*_1_ < *r*_2_; *r* = tumor diameter in mm/2)

#### Estimation of lysosomal marker enzymes

The enzymatic activity of acid phosphatase and cathepsin-D was evaluated as reported previously,^31,32^ and values were expressed as phenol/protein min per mg and tyrosine/protein min per mg, respectively. β-d-Glucuronidase was assayed by the method of Kawai and Anno,^33^ and the activity was expressed as *p*-nitrophenol (μ per moles) formed per min per mg protein.

#### Measurement of caspase-3, -8, and -9 activities

Caspase activation is a common apoptotic mechanism by which anticancer agents induce apoptosis. Hence, the level of caspase activation was determined for PTX- and 7-HC-loaded SLN system. The activities of caspase-3, -8, and -9 were determined using a caspase colorimetric assay kit according to the manufacturer's recommended protocol (R and D Systems Inc., USA). Spectrophotometric detection by the chromophore *p*-nitroanilide (*p*NA) assay was used for the cleavage from the labeled substrate that recognizes an optimal tetrapeptide sequence of the individual activation sites. Briefly, tissue homogenates were washed with ice-cold PBS, lysed with 50 μL of cold lysis buffer and incubated on ice for 10 min. Protein concentrations of the homogenates were assessed by the Bradford method, and 200 μg of protein was diluted in 50 μL lysis buffer solution. In addition to that, 50 μL of 2× reaction buffer (containing 10 mM DTT) and 4 mM DEVD-*p*NA substrate were added to each sample well. After incubation at 27 °C for 2 h, samples were read at 405 nm using a microplate reader (BioTek, USA). Changes in caspase activity were determined by comparing these results with the level of control.

#### Histopathological analysis

The evaluation of mammary and liver tissues preserved in formaldehyde (10%) and embedded in paraffin was conducted through microscopic examination. The tissues were separated at 4–5 μm thickness *via* a semi-automated microtome and consequently stained with hematoxylin/eosin. Sections were viewed under a microscope (20× magnification).

### Statistical analysis

The results were analyzed using a single way of variance, and whole results were obtained as mean ± standard deviation (SD). All experimental work was performed in triplicate. Results with *P < 0.05 were considered statistically significant.

## Results

### Synthesis of NADES-supported biotin-conjugated solid–liquid polymer

For the formulation of the NADES, 4-hydroxyprolinebetaine and PB were isolated from *Citrus limon* peels and identified by HPLC, ^1^H-NMR, ^13^C-NMR, and FT-IR spectroscopy and the spectroscopic results were compared with those of previous reports.^34^ The purity of 4-hydroxyprolinebetaine and PB was identified by HPLC with 70 : 30 and 50 : 50 ratios of ethyl acetate/methanol solvent, respectively. The HPLC trace of the 70 : 30 ratio fractions revealed two peaks, one major peak and one minor peak. The major peak noted at a retention time (RT) of 25.6 corresponds to PB, and the minor peak observed at a RT of 23.6 corresponds to 4-hydroxyprolinebetaine (ESI Fig. 2†). These peaks were confirmed by standards and previously reported literature.^34^ Similarly, in the ethyl acetate/methanol 50 : 50 ratio fraction, the major peak was noted at a RT of 24.9, corresponding to PB.

**Scheme 1:**
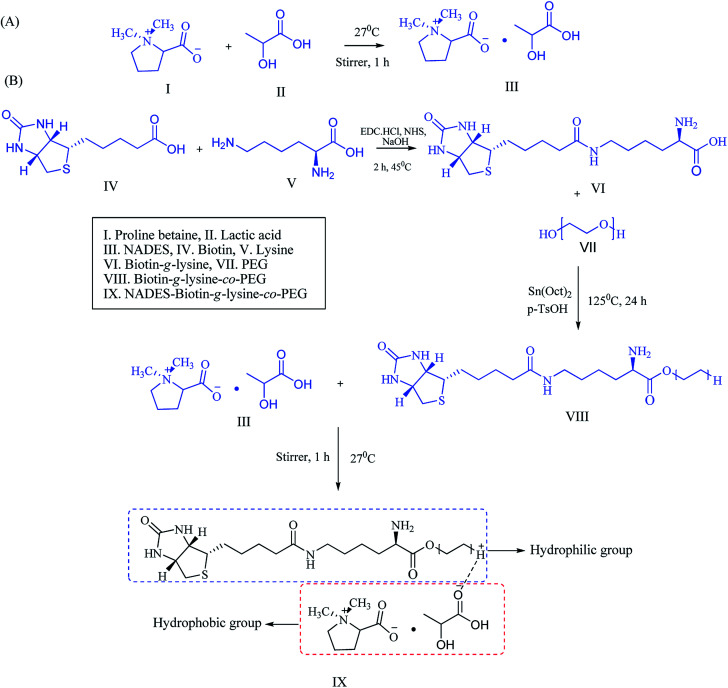
Schematic representation of (A) NADES synthesis and (B) synthetic route of the biotin-conjugated solid–liquid polymer.

The secondary metabolites PB and LA were used for the synthesis of NADESs with various ratios of 1 : 1, 1 : 2, 1 : 3, and 2 : 1. Ratios of 1 : 2 and 1 : 3 formed as a transparent solution, and the physicochemical properties were characterized, including the melting point and density of the solvents. NADES was created into exact molar ratios of the components to reach the eutectic point (Scheme 1A). NADES-based solid–liquid polymeric nanocarrier was synthesized by the interaction of free amine groups of the biotin-*g*-lysine-*co*-PEG polymer and –OH groups of the PB–LA solvent. The synthetic route of the NADES-based biotin-*g*-lysine-*co*-PEG polymer is given in Scheme 1A.

### Density analysis

Solvent density plays a critical role in chemical synthesis, and it is an essential physical property. In drug carrier synthesis using ILs, DES, and NADES, density plays a significant role in the formation of drug delivery systems (DDSs).^29^ The densities of the NADES-1 : 1, NADES-1 : 2, NADES-1 : 3, and NADES-2 : 1 solvents are mentioned in Table 2. The NADES density values ranged from 1.1571 to 1.1713 g cm^−3^ at room temperature (27 °C). NADES-1:3 had the maximum density (1.1713 g cm^−3^) at 27 °C. As the concentration of LA increased, simultaneously the molecular kinetic energy also increased because of the vibration of the atoms which were available in the NADES. It is seen that since the mobility of the NADES improved, the densities of the solvent decreased.

**Table 2:** The density of the NADESs^a^

Code	Composition	Temperature (°C)	Density (g cm^−3^)	Observation (RT)
1	PB : LA (1 : 1)	27	1.1571	Semisolid
2	PB : LA (1 : 2)	27	1.1425	Transparent liquid
3	PB : LA (1 : 3)	27	1.1713	Highly viscous
4	PB : LA (2 : 1)	27	1.1358	Semisolid

a PB, prolinebetaine; LA, lactic acid; RT, room temperature.

### NMR analysis

The interaction of the PB : LA-based NADES formation was examined by using ^1^H- and ^13^C-NMR to confirm the structure. The HBA of PB characteristic signal of ^1^H values: 4.23 (t, CH), 3.05 (s, 2CH_3_), 3.00 (q, CH_2_), 2.01 (p, CH_2_), 1.83 (q, CH_2_) and ^13^C NMR values: 185, 93, 55, 43, and 28 (ESI Fig. 3†). The HBD of LA showed ^1^H NMR values: 4.67 (s, OH), 4.11 (s, OH), 1.47 (d, 3H), 1.11 (1, 1H), and ^13^C NMR values: 185, 70, 25 (ESI Fig. 4†). After the interaction of PB : LA (NADES-1:2) ^1^H values: 10.73 (s, 1H), 5.21 (q, CH_2_), 4.21 (q, CH_2_), 3.36 (s, 2CH_2_), 3.42 (q, CH_2_), 2.56 (s, 1H), 1.81 (p, CH_2_), 1.79 (p, CH_2_), 0.8 (s, CH_2_) and ^13^C NMR values: 170, 89, 77, 75, 58, 28, and 26 (ESI Fig. 5†). In ESI Fig. 6,† the formation of NADES-1:3 characteristic signal of the ^1^H NMR values: 10.71 (s, 1H), 6.51 (q, CH_2_), 5.51 (q, CH_2_), 4.45 (s, 2CH_2_), 4.01 (q, CH_2_), 4.00 (s, 1H), 2.71 (p, CH_2_), 2.62 (p, CH_2_), 2.01 (s, CH_2_) and ^13^C NMR values: 173, 93, 79, 78, 56, 27, and 24. The spectral results show that the hydrogen bonding of (O–H^+^⋯−O) group is indicative of interaction with HBA and HBD in NADES formation (Fig. S2C†). The characteristic ^1^H signals of NADES were shifted downfield when compared with free PB and LA, which is a result of excellent hydrogen bond interaction of NADES.^35^

### FT-IR analysis

**Fig. 1:**
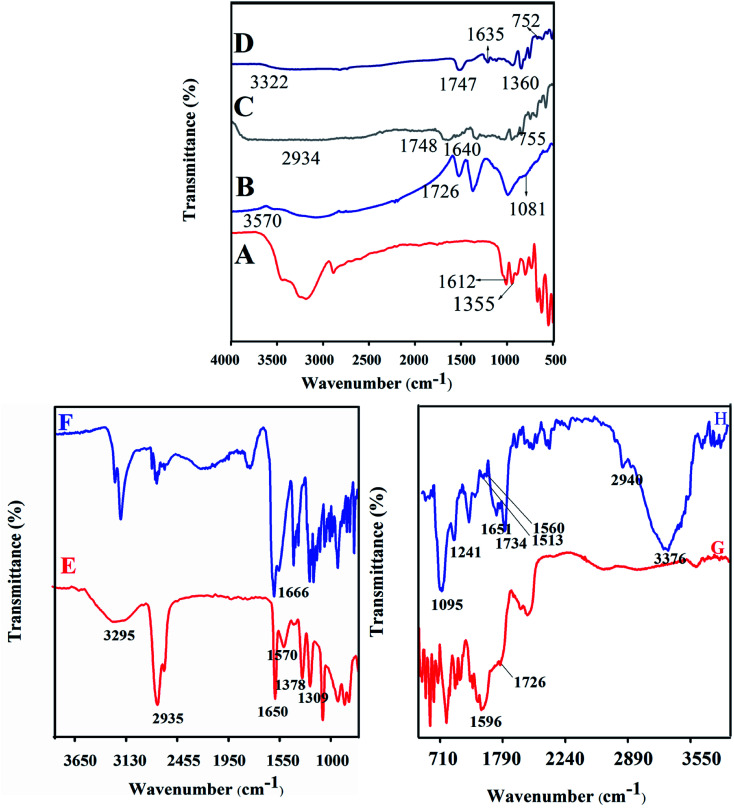
FT-IR spectra of (A) prolinebetaine, (B) lactic acid, (C) NADES-1:2, (D) NADES-1:3, (E) biotin, (F) biotin-*g*-lysine, (G) biotin-*g*-lysine-*co*-PEG, and (H) PTX- and 7-HC-loaded NADES-based biotin-conjugated solid–liquid polymer (PTX- and 7-HC-loaded nanocarrier).

PB, LA, NADES, biotin-*g*-lysine-*co*-PEG polymer, and PTX- and 7-HC-loaded NADES-based biotin-conjugated solid–liquid polymer nanocarrier were characterized by FT-IR spectroscopy. The results are shown in Fig. 1. Fig. 1A shows the absorption bands of PB functional groups at 1355 cm^−1^ (–CH stretching vibration) and 1612 cm^−1^ (carbonyl stretching vibrations). Fig. 1B shows the LA moiety characteristic peaks at 3570, 1726, and 1081 cm^−1^ corresponding to the OH, C–O, and C–C group vibration peaks. Fig. 1C and D present the typical spectra of NADES-1:2 and NADES-1:3 solvents. The COO–, C–O, –CH and C–C bands were observed at 1360 cm^−1^, 1635 cm^−1^, 1646 cm^−1^, 1638 cm^−1^, 755 and 752 cm^−1^.^15,16^ Broad peaks were observed at 2934 and 3322 cm^−1^, which indicates that the hydroxyl group (OH) was involved in strong intramolecular hydrogen bonding during NADES formation. Also, the new characteristic peaks of NADES confirm the interaction of LA and PB moiety. As shown in Fig. 1E, the peaks at 3295 cm^−1^ (O–H), 2935 cm^−1^ (C–H), 1570 cm^−1^ (N–H), 1378 cm^−1^ (C–C), 1690 cm^−1^ (C=O), and 1378 cm^−1^ (C–O) are revealed by biotin molecule. Fig. 1F shows a strong peak at 1666 cm^−1^ corresponding to amide bonds and represents the amide bond of the biotin grafted lysine (biotin-*g*-lysine).^17,36^ The FT-IR spectrum of biotin-*g*-lysine-*co*-PEG (biotin-conjugated solid–liquid polymer) (Fig. 1G) demonstrates characteristic peaks at 1596 and 1726 cm^−1^, which represent the amide and ester stretching vibration of PEG and biotin-*g*-lysine, respectively.^37^ The characteristic peaks at 1095, 1241, 1513, 1560, 1651, 1734, 2940, and 3376 cm^−1^ show a strong hydrophobic interaction between nanocarriers and drugs (PTX and 7-HC). All the characteristic peaks confirm the synthesis of PTX- and 7-HC-loaded NADES-based biotin-conjugated solid–liquid polymer nanocarrier (Fig. 1H).

### XRD, Raman spectroscopy and TGA

**Fig. 2:**
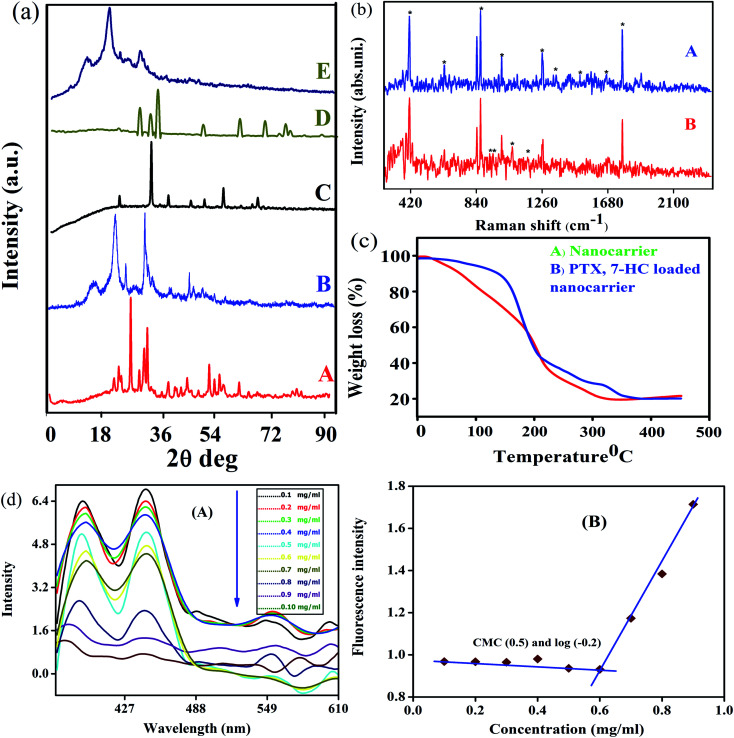
(a) XRD patterns of (A) biotin-conjugated solid–liquid polymer, (B) NADES-based biotin-conjugated solid–liquid polymer, (C) PTX, (D) 7-HC, and (E) PTX- and 7-HC-loaded nanocarrier. (b) Raman spectra of (A) nanocarrier and (B) PTX- and 7-HC-loaded nanocarrier. (c) TGA measurements for (A) nanocarrier and (B) PTX- and 7-HC-loaded nanocarrier. (d) Fluorescence spectra of micelles (A) intensity ratio *I*_1_/*I*_3_ of pyrene as a function of nanocarrier concentration, and (B) the CMC in an aqueous medium is determined to be about 0.6 mg mL^−1^.

The XRD patterns and Raman spectra of biotin-*g*-lysine-*co*-PEG polymer, NADES-based biotin-conjugated solid–liquid polymer, and PTX- and 7-HC-loaded nanocarrier were studied. The XRD pattern of biotin-conjugated solid–liquid polymer nanocarrier exhibited peaks at 2*θ* values of 23.11, 25.6, 26.5, 27.8, 29.9, 31.56, 43.90, 44, 49.32, 50.1, 53.5 and 56.0°, indicating the grafted polymer is semi-crystalline (Fig. 2a(A)). As shown in Fig. 2a(B), the intensity of the characteristic peaks was decreased due to the interaction between NADES biotin-*g*-lysine-*co*-PEG polymer in the formation of SLN, and the 2*θ* values are 15.41, 22.47, 25.71, 31.70, and 45.31°. PTX shows multiple peaks at 2*θ* values of 23, 35, 39.01, 41.25, 44.56, 47.21, and 66° due to its crystalline nature (Fig. 2a(C)).^38^ The XRD pattern of 7-HC exhibited peaks appearing at 2*θ* values of 28.15, 30.15, 31.39, 32.20, 33.83, 44.37, 46.00, 54.92, 62.23, 65.83, 67.52, and 69.97° (Fig. 2a(D)).^39^ After encapsulation of both drugs in the SLN system, the XRD pattern shows broad, less intense peaks, with a minor shift. This indicates that the drug-encapsulating SLN system has excellent interactions with a semi-crystalline nature (Fig. 2a(E)). Generally, a semi-crystalline system has a good interaction with biological liquids, which may be fitting for penetration through tissue layers and might provide an excellent diffusion property for the nanocarriers with drug molecules. Since the nature of the PTX- and 7-HC-loaded SLN system allows for interaction with the cell membranes, these materials have been used.

Raman spectra were investigated to study the interaction between PTX and 7-HC and SLN (Fig. 2b). The bands at 1126 cm^−1^ are assigned to the C–N stretching vibration mode of PB, and the band at 865 cm^−1^ is attributed to the strong C–COO bending mode.^26^ The bands at 1319 cm^−1^, 1371 cm^−1^, and 1069 cm^−1^ are attributed to the strong CH_3_ stretching mode. The band at 1680 cm^−1^ was assigned to the ester vibration mode.^40^ The characteristic vibration bands at 1680 cm^−1^ corresponded to the amide stretching vibration modes, as shown in Fig. 2b(A). Fig. 2b(B) shows the Raman spectrum of PTX- and 7-HC-loaded nanocarrier and vibration bands appeared at 278, 293, 309, 340, 366, 392, 926, 952, and 1066 cm^−1^. This reveals the durable hydrophobic nature and hydrophobic interaction between nanocarrier and drugs.

The thermal stability and decomposition of the unloaded and PTX- and 7-HC-loaded nanocarrier were characterized by TGA. Fig. 2c reveals PTX- and 7-HC-loaded nanocarrier degradation occurs as a multistep process compared with the nanocarrier alone. Thermal stability is a powerful force of molecules for bond energy and interactions. The TGA curve shows a slight decrease in the range of 25–35 °C and corresponds to the elimination of moisture in the nanocarrier (Fig. 2c(A)). The 2nd stage of weight reduction is from 150 to 165 °C due to CO and CO_2_,^41^ and the third stage of weight loss from 320 to 350 °C is related to the decomposition of ester and amide linkages. In contrast, from the weight loss of PTX- and 7-HC-loaded nanocarrier, it was found to have a higher thermal stability compared to the unloaded nanocarrier. At a temperature of 210 °C, the mass had reduced by 85% in the carrier, followed by a gradual reduction of weight at 400–500 °C (Fig. 2c(B)).

### Determination of self-assembly of carrier formation

The assistance of NADES enabled the synthesis of the NADES-based biotin-conjugated solid–liquid polymer nanocarrier through self-assembly. Here, the biotin-conjugated solid–liquid polymer nanocarrier acted with hydrophilic character and NADES with hydrophobic character, and it was a self-assembled from a solid–liquid polymeric system by dialysis. The self-assembling of the NADES-based biotin-conjugated solid–liquid polymer system was confirmed through critical nanocarrier concentration using pyrene as a fluorescent probe. The first and third shift of the NADES-based biotin-conjugated solid–liquid polymer nanocarrier (0.1 mg mL^−1^) was found in the range of *I*_390_/*I*_445_, respectively. The final concentration of the NADES-based biotin-conjugated solid–liquid polymer nanocarrier (0.9 mg mL^−1^) was observed at *I*_377_/*I*_442_. The shifting of the higher region to lower one by changing the concentrations confirmed the amphiphilic polymer changes (Fig. 2d). The CMC value of NADES-based biotin-conjugated solid–liquid polymer nanocarrier was identified in the aqueous medium and observed at 0.6 mg mL^−1^.

### Surface morphological analysis

**Fig. 3:**
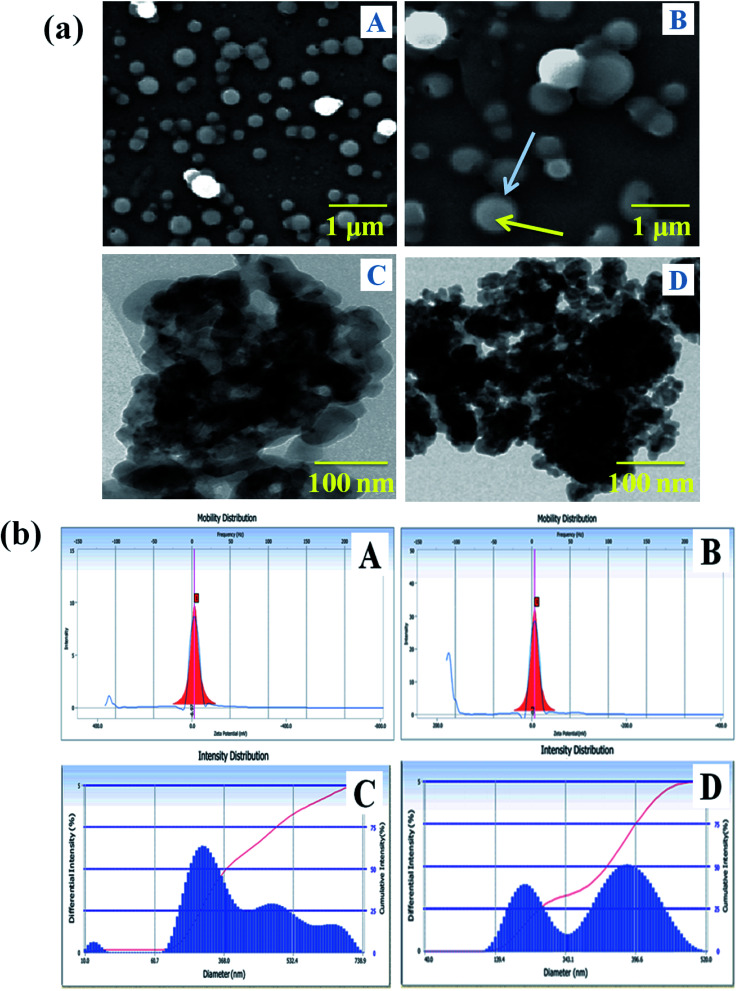
(a) SEM images of (A) nanocarrier and (B) PTX- and 7-HC-loaded nanocarrier. TEM images of (C) nanocarrier and (D) PTX- and 7-HC-loaded nanocarrier. The blue arrow indicates the hydrophilic outer layer, and the yellow arrow indicates the hydrophobic inner layer. (b) Zeta potential of (A) nanocarrier and (B) PTX- and 7-HC-loaded nanocarrier, and particle size of (C) nanocarrier and (D) PTX- and 7-HC-loaded nanocarrier.

The size determination and morphological characterization of the nanocarrier were conducted by SEM and TEM instrumental techniques (Fig. 3a). The surface feature of the carrier system can enhance the bio-distribution and pharmacokinetics of drugs in biological systems, and it has a vital role in the treatment of the diseases.^42^Fig. 3a(A) shows that the nanocarrier is spherical, its size is increased with the incorporation of drugs and it is semi-crystalline. The SLN particle observed in the SEM image showed that the NADES liquid was covering to stabilize of the solid particle, and the solid portion was highly dense in the drug-loaded SLN system (Fig. 3a(B)). Furthermore, the PTX- and 7-HC-loaded nanocarrier was spherical, and the size of the particle increased due to the charged drug molecules. Fig. 3a(C and D) show representative TEM images of the nanocarrier, the particles of which are spherical with a mean particle size of 200–300 nm; this correlates well with the SEM images.

### Particle size analysis and zeta potential determinations

The surface charge and particle size of the unloaded and PTX- and 7-HC-loaded SLN system were tested using DLS, and the results are presented in Fig. 3b. The zeta potential was observed at −3 ± 0.5 mV and −0.2 ± 0.5 mV for unloaded and PTX- and 7-HC-loaded SLN systems, respectively. From these observations, drug-loaded SLN had enhanced stability over the unloaded carrier, due to the interaction of the drugs and carrier molecules. The observed zeta potential was nearly zero (Fig. 3b(A and B)). The surface charge of the nanocarrier has a meaningful relevance for biological applications for stability, interaction with cells, and also tissues.^43^ The size distribution of the nanocarrier was observed in the range of ≈240 nm for the unloaded and ≈290 nm for the loaded carrier. This indicated that molecule size expanded as PTX and 7-HC were incorporated in the nanocarrier (Fig. 3b(C and D)).

### *In vitro* release

**Fig. 4:**
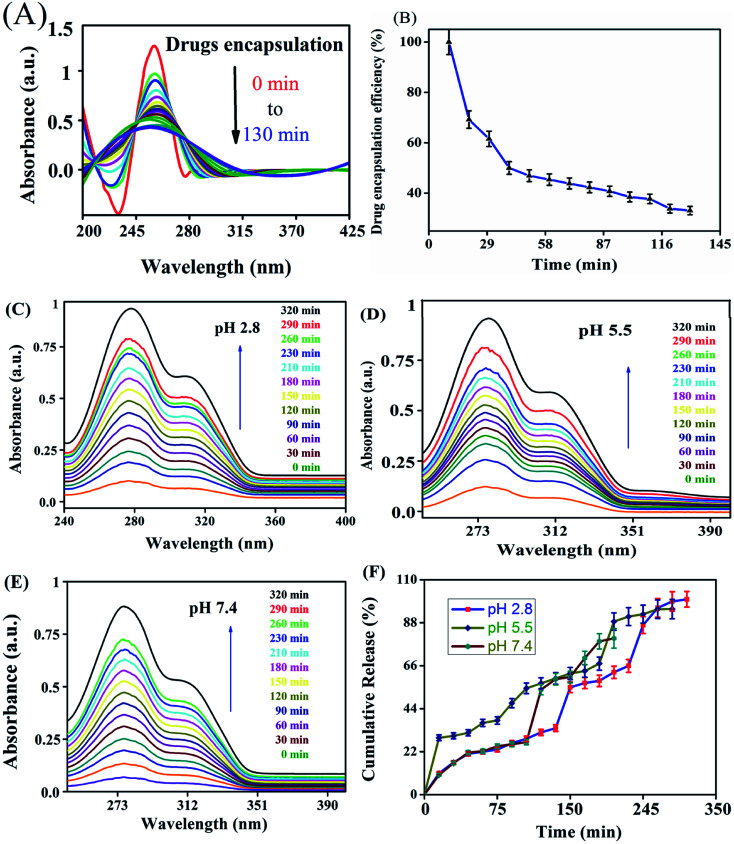
UV-visible spectroscopy showing (A) encapsulation efficiency and (B) percentage encapsulation efficiency of PTX- and 7-HC-loaded nanocarrier. *In vitro* drug discharge status of PTX- and 7-HC-loaded nanocarrier at (C) pH 2.8, (D) pH 5.5, and (E) pH 7.4. (F) Cumulative drug release pattern over 320 min.

The *in vitro* drug release profiles of anticancer drugs discharged from the nanocarrier were investigated for solutions at different pH by UV-visible spectroscopy (Fig. 4). The variation of drug release at different pH indicates that the carrier has pH-dependent drug release. At an acidic pH, a burst in drug release was observed when compared with other pH values. At pH 2.8, 27.47% drug release was observed in the first 30 min. On the other hand, at pH 7.4, only 8.79% was released from the carrier. The drug was released from the nanocarrier for up to 320 min, with a maximum drug release at an acidic pH of 2.8 (97.0%) compared with a pH of 7.4 (89%) and pH 5.5 (92%) within 320 min. Burst releases followed by the increasing drug discharge pattern of the drugs from the carrier in a strongly acidic solution could be due to the amine group and ester easily protonating the nanocarrier in a strongly acidic environment compared with a nearly neutral pH solution.

### *In vitro* cell cytotoxicity

**Fig. 5:**
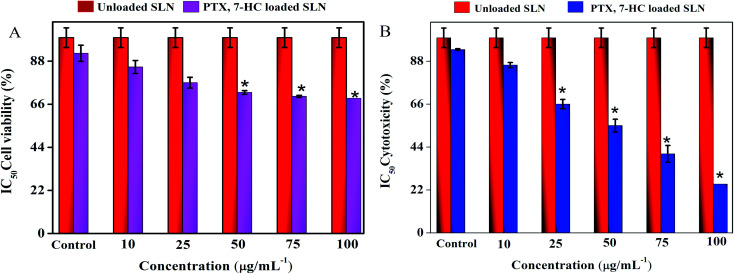
(A) The cell viability of PTX- and 7-HC-loaded SLN-treated normal cell line. (B) The cytotoxicity of PTX- and 7-HC-loaded SLN against MDA-MB-231 cancer cell line.

The cytotoxic effect of SLN systems was estimated using a normal fibroblast cell line (L929) and a breast cancer cell line (MDA-MB-231) through the MTT assay for 24 h with various concentrations. The treatment of unloaded SLN and PTX- and 7-HC-loaded SLN system retains cell viability in the normal fibroblast cell line (ESI Fig. 8A†), with quantitative cell viability being shown in Fig. 5A. ESI Fig. 8B† shows the viability of MDA-MB-231 cells treated with unloaded and PTX- and 7-HC-loaded SLN. Viability decreased at a concentration of 100 μg mL^−1^, *i.e.*, increased cell death was noticed when compared with the nanocarrier at lower concentrations; the cell cytotoxicity value of various concentrations is presented in Fig. 5B.

### Cellular uptake studies

**Fig. 6:**
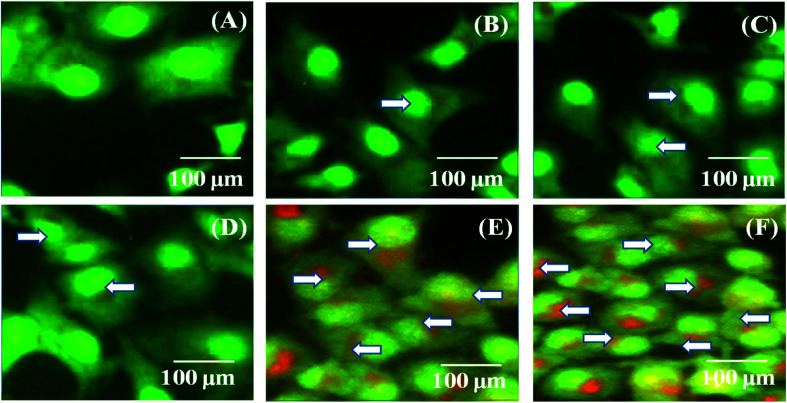
Confocal images of MDA-MB-231 cancer cells incubated with PTX- and 7-HC-loaded SLN system at 27 °C for (A) 1 h, (B) 5 h, (C) 10 h, (D) 15 h, (E) 20 h, and (F) 24 h. White arrows indicate the presence of PTX- and 7-HC-loaded SLN around nuclei.

MDA-MB-231 breast cancer cells were cultured and treated with the PTX- and 7-HC-loaded SLN system for 24 h (Fig. 6). Fluorescence images were observed after 1 h, 5 h, 10 h, 15 h, 20 h, and 24 h. As the treatment time increased, fluorescent intensities increased, which is due to a higher uptake of PTX- and 7-HC-loaded carriers *via* receptor-mediated endocytosis. The fluorescence signal was very strong, and co-localization around the nucleus (white arrow) was observed at 20 h and 24 h in the PTX- and 7-HC-loaded SLN system-treated samples.

### *In vivo* studies

#### Lysosomal enzymes

**Fig. 7:**
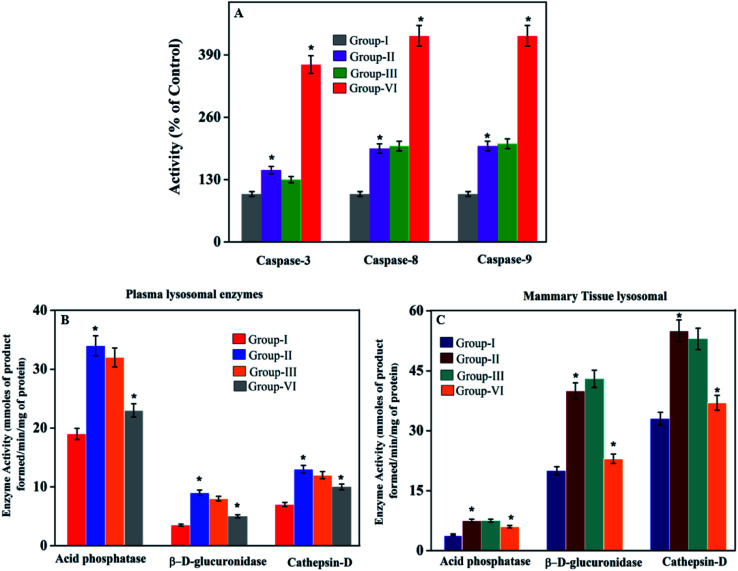
(B and C) Effect of PTX- and 7-HC-loaded nanocarrier on lysosomal marker enzymes. Group I, control; group II, cancer induced; group III, SLN unloaded treated; group IV, SLN loaded treated. Each value is expressed as the mean ± SD for six rats in each group. Significance compared to group I is shown as *P < 0.05. (A) Effect of PTX- and 7-HC-loaded nanocarrier on caspase-3, -8, and -9. Group I, control; group II, cancer induced; group III, SLN unloaded treated; group IV, SLN loaded treated. Each value is expressed as the mean ± SD for six rats in each group. Significant difference as compared to group I shown as *P < 0.05.

Fig. 7A presents the levels of lysosomal marker enzymes such as acid phosphatase, β-d-glucosaminidase, and cathepsin-D in plasma and mammary tissue of control and treated rats.

#### Effects of PTX- and7-HC-loaded SLN on caspase-3, -8 and -9 activities

To elucidate cell death induced by PTX- and 7-HC-loaded SLN, the possible involvement of caspases was assessed (Fig. 7B).

#### Histological studies

**Fig. 8:**
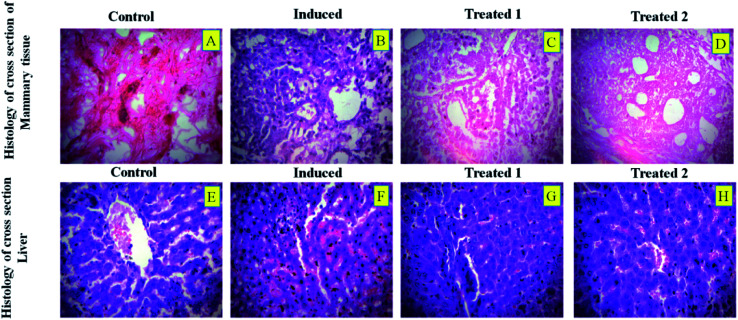
Histological sections of mammary tissue of DMBA-induced breast cancer in rats from (A) control, (B) cancer induced, (C) SLN unloaded treated, and (D) SLN loaded treated. Liver sections of DMBA-induced breast cancer in rats from (E) control, (F) cancer induced, (G) SLN unloaded treated, and (H) SLN loaded treated.

The histological sections of breast tissue and liver tissue of DMBA-induced breast cancer in rats treated with 5 mg per g b.wt of PTX- and 7-HC-loaded SLN are shown in Fig. 8. In both the liver and the mammary tissue sections, the loaded SLN-treated animals showed normal and healthy architecture when compared to the other groups.

## Discussion

The SLN size was chosen based on tissue penetration, prolonged persistence in the bloodstream thereby avoiding renal filtration, and also better effectiveness for cancer therapy. As a result, the swelling behavior increased as the pH decreased. Moreover, the NADES-based biotin-conjugated solid–liquid polymer, after arriving at the targeted cancer cells, will be degraded by lysosomal enzymes that are abundant in cancer cells. The primary role of these enzymes is the degradation of ester and amide bonds. In this system, the drug carrier has both amide and ester bonds. The amide bonds found in the NADES will allow the lysosomal enzymes to simply degrade the biotin-conjugated solid–liquid polymer in cancer cells.^41^ Thus, it will help the release of drugs into cancer cells. The cell cytotoxicity effect of the PTX- and 7-HC-loaded SLN is due to a high negative surface charge that eases the binding on the surface of cancer cells, thereby damaging the cell walls and altering adenosine triphosphate (ATP) production. Cell wall damage is the most significant determinant of the cytotoxic activity due to the strong electrostatic interaction involving the negatively charged polymer carrier and the positively charged cell membrane.^44^ Based on the cell uptake properties of the drug-loaded polymeric micelles, this suggests that PTX and 7-HC present in the nanocarrier interact with DNA and damage the nuclear membrane of cancer cells.^38^ From this, it could be seen that there are changes in the tumor volume and body weight, which are shown in Table 3. In cancer rats, the body weight was decreased (*P* < 0.05) as compared to the control rats. In PTX- and 7-HC-loaded SLN system-treated rats, the body weight was notably recovered and also the tumor size significantly reduced as compared with the cancer-induced and unloaded nanocarrier-treated rats. The lysosomal enzyme activities were significantly elevated (plasma and mammary tissue (*P* < 0.05)) in group II animals when compared with the controls. This may be due to the abnormal release of lysosomes in cancer conditions. Also, the elevated levels could reflect the increased synthesis and secretion of lysosomal enzymes by tumors.^35^ Due to the treatment with PTX- and 7-HC-loaded SLN system in group IV animals, the enzyme levels decreased to near normal.

**Table 3:** Effect of nanocarriers on body weight and tumor volume in control and experimental rats^a^

Body weight	Group I	Group II	Group III	Group IV
Initial	199.5 ± 8.26	197.8 ± 2.99	198.6 ± 5.39	195.6 ± 5.81
Final	239.5 ± 4.33	210.1 ± 3.98*	212.7 ± 4.85*	243.2 ± 9.21
Tumor volume (mm^2^)	—	4502 ± 380*	4289 ± 403*	2954 ± 335

a Group I, control; group II, cancer induced; group III, SLN unloaded treated; group IV, SLN loaded treated. Each value is expressed as the mean ± SD for six rats in each group. Significance as compared to group I shown as *P < 0.05.

In most cases, different anticancer agents eventually mediate a common apoptotic pathway through the activation of caspases. The enzymatic activity of caspase-3, -8, and -9 in the PTX- and 7-HC-loaded SLN-treated rats increased significantly. Otherwise, group II is a cancer-induced cell line. After that, the induced cell line was incubated with polymeric micelles, which gradually increased in group III due to the SLN's functional group activity in the cancer cell line. On the other hand, the caspase levels in the SLN-treated group remain unchanged. This reflects that the treatment with PTX- and 7-HC-loaded SLN leads to a strong cleavage of the biotin targeted molecules. However, the targeted drug delivery of PTX- and 7-HC-charged SLN to tumor cells excellently enhanced the apoptosis-induced cell death. The histological sections of PTX- and 7-HC-loaded SLN-treated rats reveal the presence of atrophy of seromucous glands with surrounding stromal fibrosis, fatty tissue with small lobules, and tumor regression. DMBA-induced rats showed tumors composed of hyperchromatic, pleomorphic, vesicular nuclei, and moderate cytoplasm arranged in nests, sheets, and acinar structures with numerous mitotic structures. Based on the results, the PTX- and 7-HC-loaded SLN can reduce side effects. Ultimately, histological studies of the SLN and PTX- and 7-HC-loaded SLN did not reveal any abnormal morphological change to the mammary tissue and liver, further showing that they are safe for therapeutic use due to the PEG corona protecting against protein absorption with highly stable SLN and PTX- and 7-HC-loaded SLN, while the PEGylated surface led to an uptake in the liver.

## Conclusion

A novel approach in advanced cancer prevention and treatment includes the use of SLN systems for anticancer drug delivery. A PTX- and 7-HC-loaded SLN was synthesized for the first time with a green solvent as a promising novel drug delivery system for the enhancement of the therapeutic index of cancer cells. The unloaded and PTX- and 7-HC-loaded SLNs were characterized in terms of size and crystalline properties. The higher toxicity of PTX- and 7-HC-loaded SLN system was observed by MTT assay, which promoted MDA-MB-231 cell death. The effect of PTX- and 7-HC-loaded SLN on cell viability was investigated with MTT assay and also by morphological observations. The decreased cell viability observed in this study could be due to direct inhibition of cell growth by PTX- and 7-HC-loaded SLN. Apoptosis was included in cell death mechanisms observed in breast cancer-induced rats. Treatment with PTX- and 7-HC-loaded SLN system elevates caspase levels that stimulate cell death mechanisms and damage tumor tissue. From these results, it is clear that the synthesized PTX- and 7-HC-loaded SLN system is highly valuable in preventing cancer cell proliferation. It is concluded that the PTX- and 7-HC-packed SLN system might be a potential candidate for breast cancer treatment and also which can improve the treatment of incurable diseases. Additional studies are suggested for estimating the effect of these nanocarriers on other cell death mechanisms.

## Abbreviations

NADES: Natural deep eutectic solvents
SLN: Solid–liquid nanocarrier
NMR: Nuclear magnetic resonance
FTIR: Fourier-transform infrared spectroscopy
TGA: Thermogravimetric analysis
XRD: Powder X-ray diffraction
DLS: Dynamic light scattering
SEM: Scanning electron microscopy
TEM: Transmission electron microscopy
PTX: Paclitaxel
7-HC: 7-Hydroxycoumarin
MDA-MB-231: M. D. Anderson and metastasis breast cancer
PB: Prolinebetaine
LA: Lactic acid
b.wt: Bodyweight
DMBA: 7,12-Dimethylbenz[*a*]anthracene
DDSs: Drug delivery systems
ILs: Ionic liquids
DESs: Deep eutectic solvents
DD water: Double-distilled water
ATP: Adenosine triphosphate
RT: Retention time

## Conflicts of interest

The authors declare that they have no competing interests.

## Supplementary Material

RA-010-D0RA03790G-s001

## References

[cit1] Brindha J., Kaushik C. (2019). Evolutionary approaches in protein engineering towards biomaterial construction. RSC Adv..

[cit2] Mehnath S., Arjama M., Rajan M., Premkumar K., Karthikeyan K., Jeyaraj M. (2020). Mineralization of bioactive marine sponge and electrophoretic deposition on Ti-6Al-4V implant for osteointegration. Surf. Coat. Technol..

[cit3] Khan F., Tanaka M., Ahmad S. R. (2015). Fabrication of Polymeric Biomaterials: A Strategy for Tissue Engineering and Medical Devices. J. Mater. Chem. B.

[cit4] Tonnesen H. H., Karlsen J. (2002). Alginate in Drug Delivery Systems. Drug Dev. Ind. Pharm..

[cit5] Palvai S., Anandi L., Sarkar S., Augustus M., Roy M., Lahiri M., Basu S. (2017). Drug-Triggered Self-Assembly of Linear Polymer into Nanoparticles for Simultaneous Delivery of Hydrophobic and Hydrophilic Drugs in Breast Cancer Cells. ACS Omega.

[cit6] Govindaraj D., Pradeepkumar P., Rajan M. (2018). Synthesis of morphology tuning multi mineral substituted apatite nanocrystals by novel natural deep eutectic solvents. Mater. Discovery.

[cit7] Graf N., Lippard S. J. (201). Redox Activation of Metal-Based Prodrugs as A Strategy for Drug Delivery. Adv. Drug Delivery Rev..

[cit8] Ishihara Y., Shimamoto N. (2006). Involvement of Endonuclease G in Nucleosomal DNA Fragmentation under Sustained Endogenous Oxidative Stress. J. Biol. Chem..

[cit9] Arguelles S. G., Serrano M. C., Gutieerrez M. C., Luisa Ferrer M., Yuste L., Rojo F., del Monte F. (2013). Deep Eutectic Solvents for the Self-Assembly of Gold Nanoparticles: A SAXS, UV−Vis, and TEM Investigation. Langmuir.

[cit10] Thai Thanh H. T., Emily H. P., Dai Hai N., Jung S. L., Ki Dong P., Nghia P. T. (2020). The Importance of Poly(ethylene glycol) Alternatives for Overcoming PEG Immunogenicity in Drug Delivery and Bioconjugation. Polymers.

[cit11] Ksenia S., Egorova S., Evgeniv G., Gordeev G., Valentine P. (2017). Biological Activity of Ionic Liquids and Their Application in Pharmaceutics and Medicine. Chem. Rev..

[cit12] Pradeepkumar P., Elgorban A. M., Bahkali A. H., Rajan M. (2018). Natural Solvent-Assisted Synthesis of Amphiphilic Co-Polymeric Nanonanocarrier for Prolonged Release of Camptothecin Delivery. New J. Chem..

[cit13] Verpoorte R. , Chemodiversity and the Biological Role of Secondary Metabolites, Some Thoughts for Selecting Plant Material for Drug Development, in Bioassay Methods in Natural Product Research and Drug Development, ed. L. Bohlin and J. G. Bruhn, Proceedings of the Phytochemical Society of Europe, Springer, Dordrecht, 1999, vol. 43, pp. 11–23

[cit14] Czaban W., Rasmussen J., Laursen B. B., Vidkjaer N. H., Sapkota R., Nicolaisen M., Fomsgaard S. (2018). Multiple Effects of Secondary Metabolites on Amino Acid Cycling in White Clover Rhizosphere. Soil Biol. Biochem..

[cit15] Ksenia S., Egorova S., Evgeniv G., Gordeev G., Valentine P. (2017). Biological Activity of Ionic Liquids and Their Application in Pharmaceutics and Medicine. Chem. Rev..

[cit16] Pradeepkumar P., Govindaraj D., Jeyaraj M., Munusamy M. A., Rajan M. (2017). Assembling of Multifunctional Latex-Based Hybrid Nanocarriers from Calotropisgigantea for Sustained (Doxorubicin) DOX Releases. Biomed. Pharmacother..

[cit17] Mukesh C., Bhatt J., Prasad K. (2014). Preparation of a NoncytotoxicHemocompatible Ion Gel by Self-Polymerization of HEMA in a Green Deep Eutectic Solvent. Macromol. Chem. Phys..

[cit18] Wang A. L., Zheng X. L., Zhao Z. Z., Li C. P., Zheng X. F. (2014). Deep Eutectic Solvent Catalyzed friedel–Crafts Alkylation of Electron-Rich Arenes with Aldehydes. RSC Adv..

[cit19] Monleon D., Morales J. M., Barrasa A., Lopez J. A., Vazquez C., Celd B. (2009). Metabolite Profiling of Fecal Water Extracts From Human Colorectal Cancer. NMR Biomed..

[cit20] Day C. R., Kempson S. A. (2016). Betaine Chemistry, Roles, and Potential Use in Liver Disease. Biochim. Biophys. Acta, Gen. Subj..

[cit21] Heinzmann S. S., Brown I. J., Chan Q., Bictash M., Dumas M. E., Kochhar S., Stamler J. (2010). Metabolic profiling strategy for discovery of nutritional biomarkers: prolinebetaine as a marker of citrus consumption. Am. J. Clin. Nutr..

[cit22] Joralemon M. J., Murthy K. S., Remsen E. E., Becker M. L., Wooley K. L. (2004). Synthesis, Characterization, and Bioavailability of Mannosylated Shell Cross-Linked Nanoparticles. Biomacromolecules.

[cit23] Kim J. H., Li Y., Kim M. S., Kang S. W., Jeong J. H., Sung Lee D. (2012). Synthesis and Evaluation of Biotin-Conjugated Ph-Responsive Polymeric Nanocarriers as Drug Carriers. Int. J. Pharm..

[cit24] Miyake Y., Yamamoto K., Morimitsu Y., Osawa T. (1997). Isolation of C-Glucosylflavone from Lemon Peel and Antioxidative Activity of Flavonoid Compounds in Lemon Fruit. J. Agric. Food Chem..

[cit25] Raghuwanshi V. S., Ochmann M., Hoell A., Polzer F., Rademann K. (2014). Deep Eutectic Solvents for the Self-Assembly of Gold Nanoparticles: A SAXS, UV−Vis, and TEM Investigation. Langmuir.

[cit26] Zhu W., Song Z., Wei P., Meng N., Teng T., Yang F., Liu N., Feng R. (2015). Y-Shaped Biotin-Conjugated Poly (Ethylene Glycol)–Poly (Epsilon-Caprolactone) Copolymer for The Targeted Delivery of Curcumin. J. Colloid Interface Sci..

[cit27] Mikhail A. S., Allen C. (2010). (Poly(ethylene glycol)-b-poly(ε-caprolactone)) Nanocarriers Containing Chemically Conjugated and Physically Entrapped Docetaxel: Synthesis, Characterization, and the Influence of the Drug on Nanocarrier Morphology. Biomacromolecules.

[cit28] Praphakar R. A., Munusamy M. A., Alarfaj A. A., Suresh Kumar S., Rajan M. (2017). Zn^2+^ Cross-Linked Sodium Alginate-G-AllylamineMannose Polymeric Carrier on Rifampicin for Macrophage Targeting Tuberculosis Nanotherapy. New J. Chem..

[cit29] Pradeepkumar P., Naresh Kumar R., Alarfaj A. A., Murugan M. A., Rajan M. (2018). Deep Eutectic Solvent-Mediated FA-g-β-Alanine-co-PCL Drug Carrier for Sustainable and Site-Specific Drug Delivery. ACS Appl. Bio Mater..

[cit30] Janas C., Mostaphaoui Z., Schmiederer L., Bauer J., Wacker M. G. (2016). Novel Polymeric Nanocarriers for Drug Delivery: Material Characterization and Formulation Screening. Int. J. Pharm..

[cit31] King J. , The hydrolases–acid and Alkaline Phosphatises, in Practical Clinical Enzymology, ed. J. C. King, D. Van Nostrand Company Ltd., London, 1965, pp. 191–208

[cit32] Sapolsky A. I., Atlman R. D., Howell D. S., Cathepsin D. (1973). Activity in Normal and osteoarthritic human cartilage. Fed. Proc..

[cit33] Kawai Y., Anno K. (1971). Mucopolysaccharide Degrading Enzymes from the Liver of Squid OmmastrephesSolaniPacificus. I. Hyaluronidase. Biochim. Biophys. Acta, Enzymol..

[cit34] Servillo L., D'Onofrio N., Giovane A., Casale R., Cautela D., Ferrari G., Castaldo D. (2018). The Betaine Profile of Cereal Flours Unveils New And Uncommon Betaines. Food Chem..

[cit35] Shinar H., Battistel M. D., Mandler M., Lichaa F., Freed berg F. L. (2014). Chemical Exchange Saturation Transfer NMR of Oligo- and Poly-Sialic Acids and the Assignment of Their Hydroxyl Groups Using Selective- and HSQC-TOCSY. Carbohydr. Res..

[cit36] Wang A. L., Zheng X. L., Zhao Z. Z., Li C. P., Zheng X. F. (2014). Deep Eutectic Solvent Catalyzed friedel–Crafts Alkylation of Electron-Rich Arenes with Aldehydes. RSC Adv..

[cit37] Chien C., Ching T., Lin A., Zhang M., Levengood S. L., Zhang M. (2016). PEG-Chitosan Hydrogel with Tunable Stiffness for Study of Drug Response of Breast Cancer Cells. Polymers.

[cit38] Zhang H., Hu H., Zhang H., Dai W., Wang X., Wang X., Zhang Q. (2015). Effects of Pegylated Paclitaxel Nanocrystals On Breast Cancer and Its Lung Metastasis. Nanoscale.

[cit39] Shtukenberg G. A., Zhu Q., Carter D. J., Vogt L., Hoja J., Schneider E., Song H. (2017). Powder Diffraction and Crystal Structure Prediction Identify Four New Coumarin Polymorphs. Chem. Sci..

[cit40] Harris W. C., Coe D. A. (1976). Vibrational spectra and structure of esters--H.* Raman spectra and potential function calculations for HC00CH3, DCOOCH3 and HCOOCD_3_. Spectrochim. Acta, Part A.

[cit41] Gheybi H., Entezami A. A. (2013). NanosizedNanocarriers Self-assembled from Amphiphilicpoly(citric acid)–poly(e-caprolactone)–poly(citric acid) Copolymers. Polym. Bull..

[cit42] Davis S. S. (1997). Biomedical Applications of Nanotechnology-Implications for Drug Targeting and Gene Therapy. Trends Biotechnol..

[cit43] Song Yang K., Mai N. V., Emily H. P., Angus P. R. J., Michael R. W., John F. Q., Nghia P. T., Thomas P. D. (2018). Elucidating the Influences of Size, Surface Chemistry, and Dynamic Flow on Cellular Association of Nanoparticles Made by Polymerization-Induced Self-Assembly. Small.

[cit44] Rajan M., Krishnan P., Pradeepkumar P., Jeyanthinath M., Jeyaraj M., Ling M. P., Arulselvan P., Higuchi A., Munusamy M. A., Arumugam R., Benelli G., Murugan K., Suresh Kumar S. (2017). Magneto-chemotherapy for cervical cancer treatment with camptothecin loaded Fe_3_O_4_functionalizedb-cyclodextrinnanovehicles. RSC Adv..

