# Supplementary material for: Natural deep eutectic solvent supported targeted solid–liquid polymer carrier for breast cancer therapy
Source: RSC Adv. 2020 Oct 7;10(61):36989–7004. doi: 10.1039/d0ra03790g (PMC9057073; doi:10.1039/d0ra03790g)
Supplement: RA-010-D0RA03790G-s001 [file RA-010-D0RA03790G-s001.pdf]

Electronic Supplementary Material (ESI) for RSC Advances.
This journal is © The Royal Society of Chemistry 2020

Supporting information

Natural Deep Eutectic Solvent Supported Targeted Solid Liquid Polymer carrier for Breast Cancer therapy

Xianfu Sun[a], Periyakaruppan Pradeepkumar[b*], Naresh Kumar Rajendran[c], Harshavardhan Shakila[d], Nicolette Nadene Houreld[c], Dunia A. Al Farraj[e], Yousif M. Elnahas[f], Nandhakumar Elumalai[g], Mariappan Rajan[b*]

[a]Department of Breast, The Affiliated Tumor Hospital of Zhengzhou University, Zhengzhou, Henan, 450008, China

[*b]Biomaterials in Medicinal Chemistry Laboratory, Department of Natural Products Chemistry, School of Chemistry, Madurai Kamaraj University, Madurai - 625021, Tamil Nadu and India.

[c]Laser Research Centre, Faculty of Health Sciences, University of Johannesburg, P.O. Box17011, Doornfontein 2028, South Africa.

[d]Department of Molecular Microbiology, School of Biotechnology, Madurai Kamaraj University, Madurai-625021, India

[e]Department of Botany and Microbiology, College of Science, King Saud University, Riyadh 11451, Saudi Arabia

[f]Research Centre, College of Medicine, King Saud University, Riyadh 11451, Saudi Arabia.

[g]Department of Biochemistry, Sri Muthukumaran Medical College and Research Institute, Chennai- 600 069, Tamil Nadu, and India.

***Corresponding author:** Mariappan Rajan, Biomaterials in Medicinal Chemistry Laboratory, Department of Natural Products Chemistry, School of Chemistry, Madurai Kamaraj University, Madurai - 625021, Tamil Nadu, India, Email: rajanm153@gmail.com

Methods

Physicochemical Characterizations

DSC analysis

Differential scanning calorimetry (DSC) was used for thermal analysis and performed on a Mettler Toledo (DSC821e/700/544/414143/5003) and then increased at regular intervals to 160°C at a heating rate of 10°C/min in an inert atmosphere of N_2.

Density

The densities of the prepared PB: LA was obtained using a gravity bottle with a standard accuracy (14.01±1 $g.cm^{-3}$). The assessed contingency of the density estimations observed was 1±1 $g.cm^{-3}$. Experimental densities calculated using equation (3):

$$\rho = \frac{m}{V} \text{........... (3)}$$

Where ρ ($g.cm^{-3}$) is the density; m is the mass of bottle; v is the volume of the substance

NMR analysis

The ^{1}H and ^{13}C spectra were recorded on Bruker (Avance) 300 MHz NMR instrument using TMS as internal standard and $CDCl_3$ and DMSO-d_6 as a solvent. Standard Bruker software was used during the spectral analysis.

FT-IR analysis

The Fourier-transform infrared (FT-IR) spectrum of PB, LA, NADES-1:1, NADES-1:2, NADES-1:3, biotin-*g*-lysine, biotin-*g*-lysine-*co*-PEG, NADES based biotin-conjugated solid-liquid polymer nanocarrier (nanocarrier), PTX and 7-HC loaded NADES based biotin-conjugated solid-liquid polymer nanocarrier was obtained by FT-IR spectroscopy(Spectrum GX-I, Perkin Elmer, Waltham, MA, USA) within the scanning range of 4000-400 cm^{-1} using the KBr pellet method.

Raman analysis

The functional group vibration of the nanocarrier, and PTX and 7-HC loaded SLNsystem were determined by Raman spectroscopy (RAM HR800) using a He-Ne laser (632 nm) with an excitation source with a power output of 17 mW.

XRD analysis

X-ray diffraction was characterized by the crystalline nature of the nanocarrier and PTX and 7-HC loaded SLN. Samples were analyzed with a PW3040/60 X pert PRO (Almelo and Netherland).

TGA analysis

The thermal nature of the nanocarrier and PTX and 7-HC loaded SLN was assessed by thermogravimetric analysis (TGA) in an N_2 atmosphere at a scanning rate of 10 k/min from 25°C to 800°C (SDTQ-600).

Particle size (DLS) analysis

Dynamic light scattering (DLS) was used to determine the particle surface charge and also size distribution of the nanocarrier and PTX and 7-HC loaded SLN system at pH 2.8. The solution was studied at 27°C in a Zetasizer (Malvern instruments DTS Ver. 4.10) equipped with a He-Ne laser (633 nm) as a light source.

Morphological analysis

The morphology of the nanocarrier and PTX and 7-HC loaded SLN system were identified by scanning electron microscopy (SEM, VEGA3SB, TESCAN, Czech Republic) and high-resolution transmission electron microscopy (HRTEM, Model TecnaiPhilipsF30, FEI Co., Hillsboro).

Results

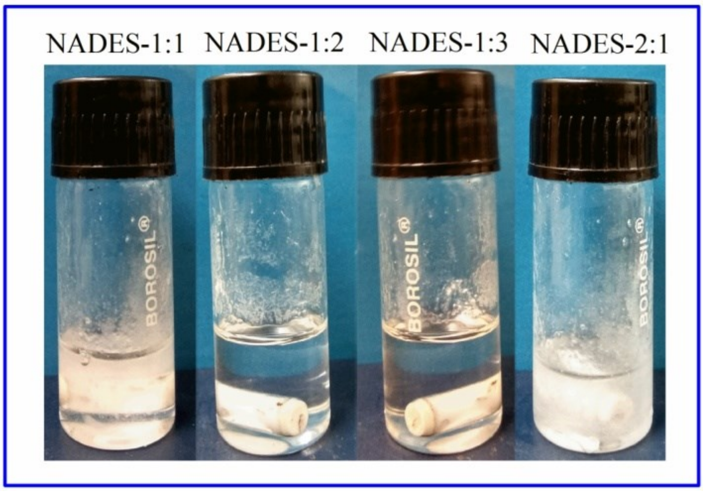

S. Figure 1. Images of natural deep eutectic solvents formation

DSC Analysis

The melting points, as per the phase transformation of NADES with PB and LA (1:2 and 1:3)mixture was analyzed using Differential Scanning Calorimeter (DSC), as shown in Figure 1 (C). Thermogram changes of NADES-1:2 isdemonstrated in Figure 2A, the peak corresponding to the melting point observed with T_M of 24˚C. As shown in Figure 1 (C) B, with an increasing amount of LA, the melting point peak shifted towards the higher temperature at T_M of 33˚C. The melting point increased with an expanding amount of LA due to the good intermolecular interactions with PB moiety. The higher mobility leads to the change of transition temperature. Finally, with the molar ratio of 1:2 NADES, the melting point is 24˚C, which is lower than 1:3 NADES and it is feasible as solvent for chemical reactions (Chemat F et al., 2016).

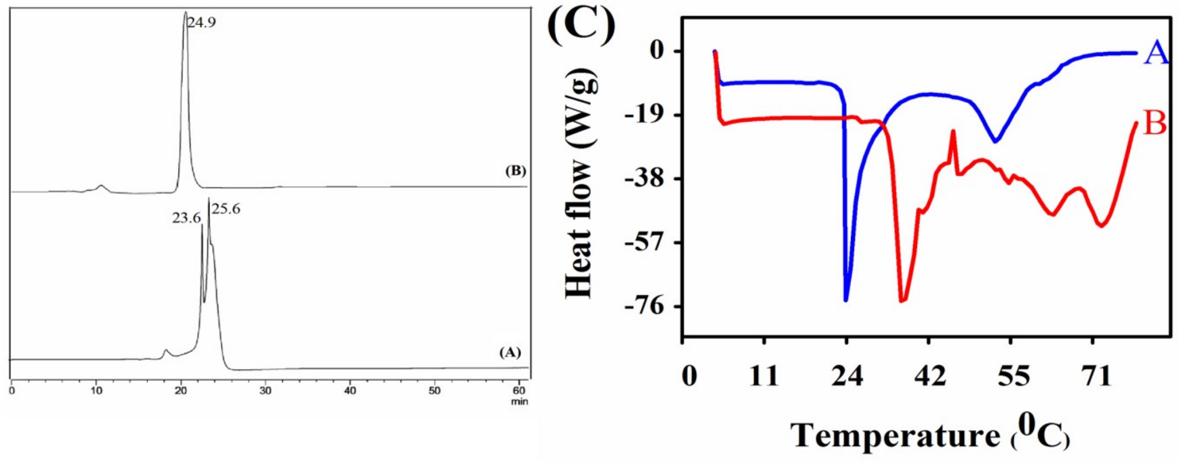

S. Figure 2. HPLC analysis of the isolation of Proline Betaine from *Citrus lemon* peels (A) ethyl acetate/methanol (30:70), and (B) ethyl acetate/methanol (50:50) values of compounds indicated in number (C) DSC thermograms of A) NADES-1:2, and B) NADES-1:3.

A

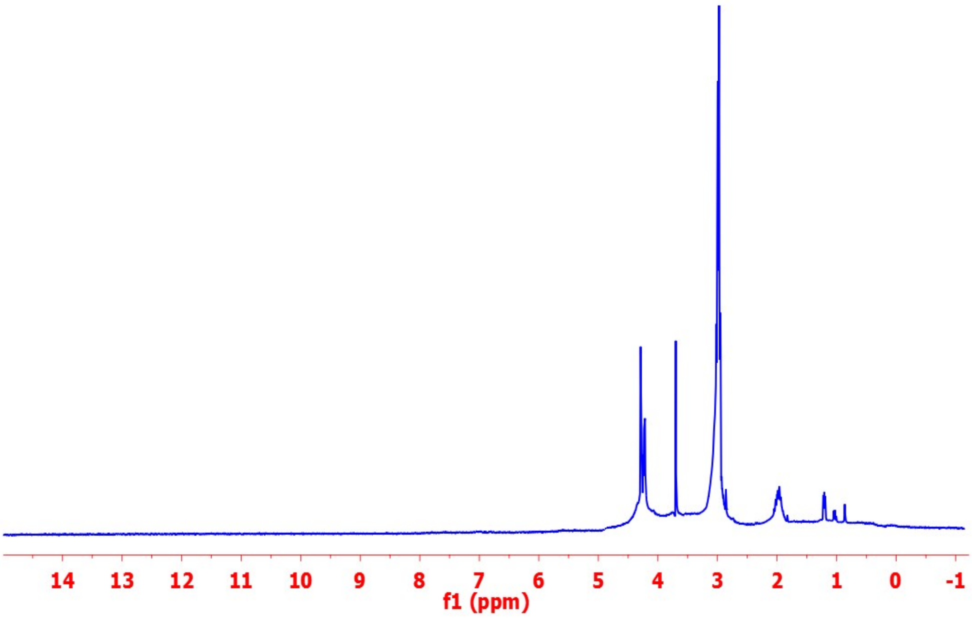

A

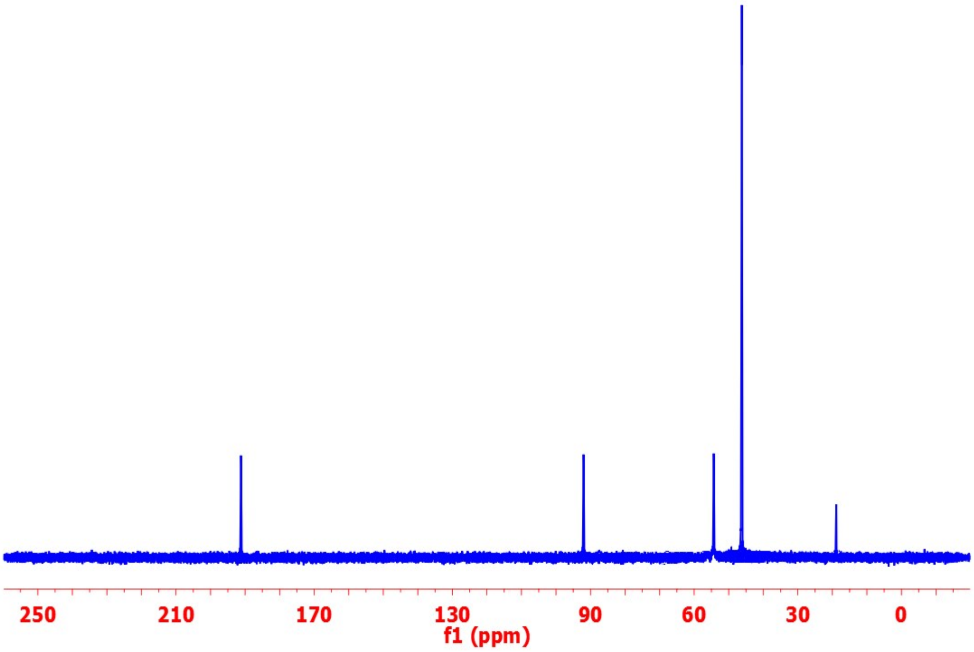

S. Figure 3. ^{1}H, ^{13}C spectrum of Prolinebetaine

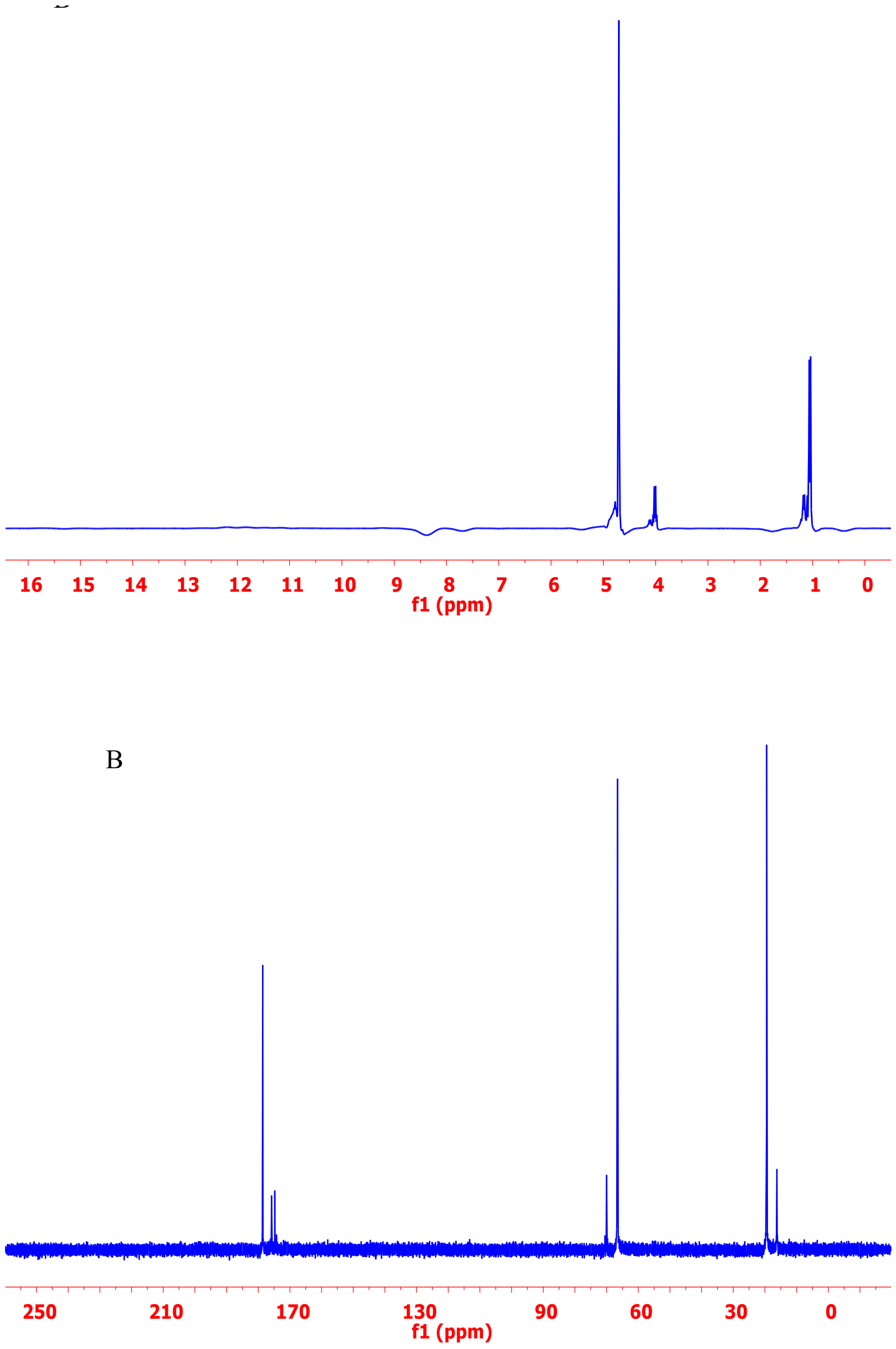

S. Figure 4. ^{1}H, ^{13}C spectrum of Lactic acid

C

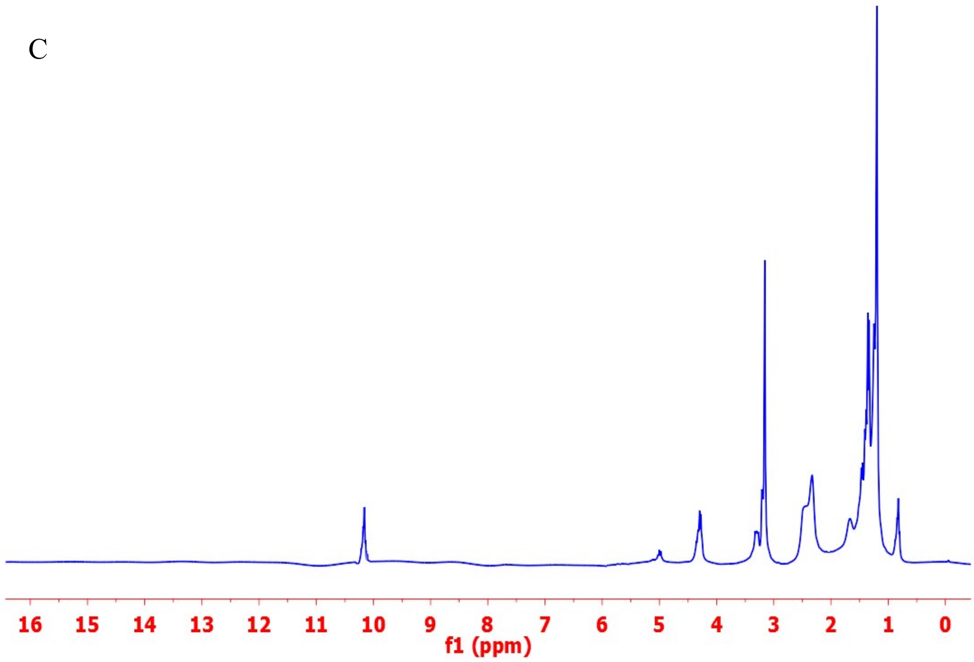

C

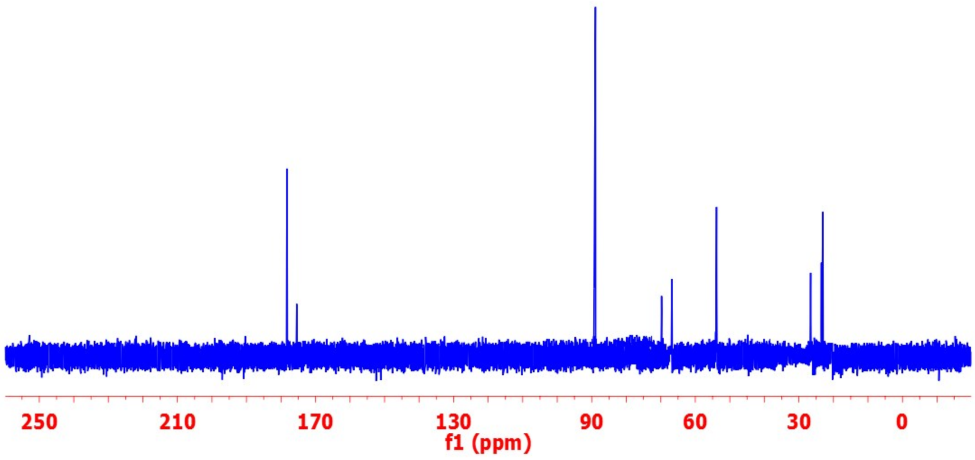

S. Figure 5. ^{1}H, ^{13}C spectrum of NADES 1:2

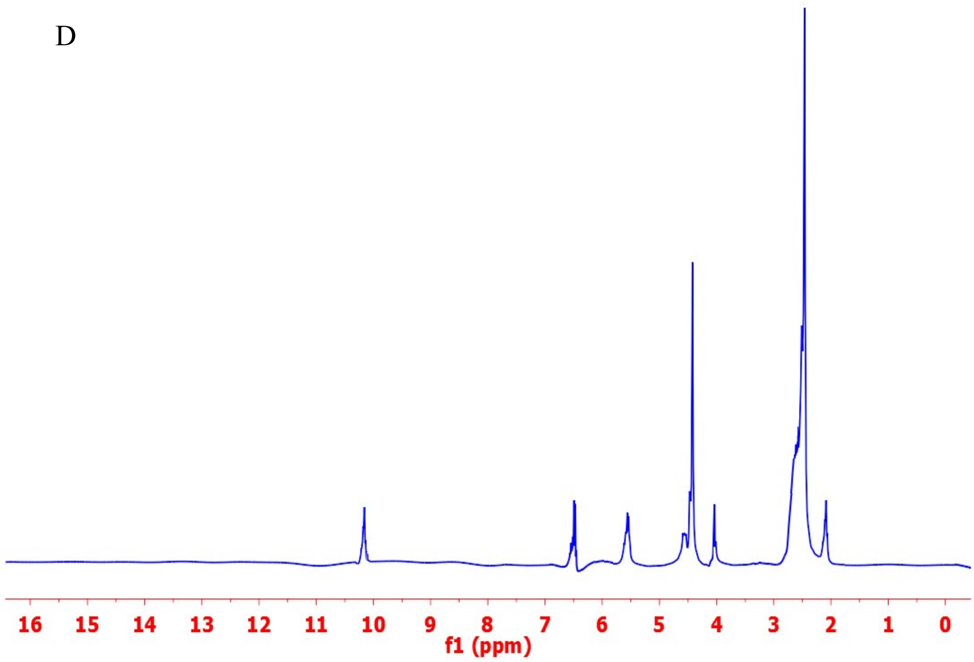

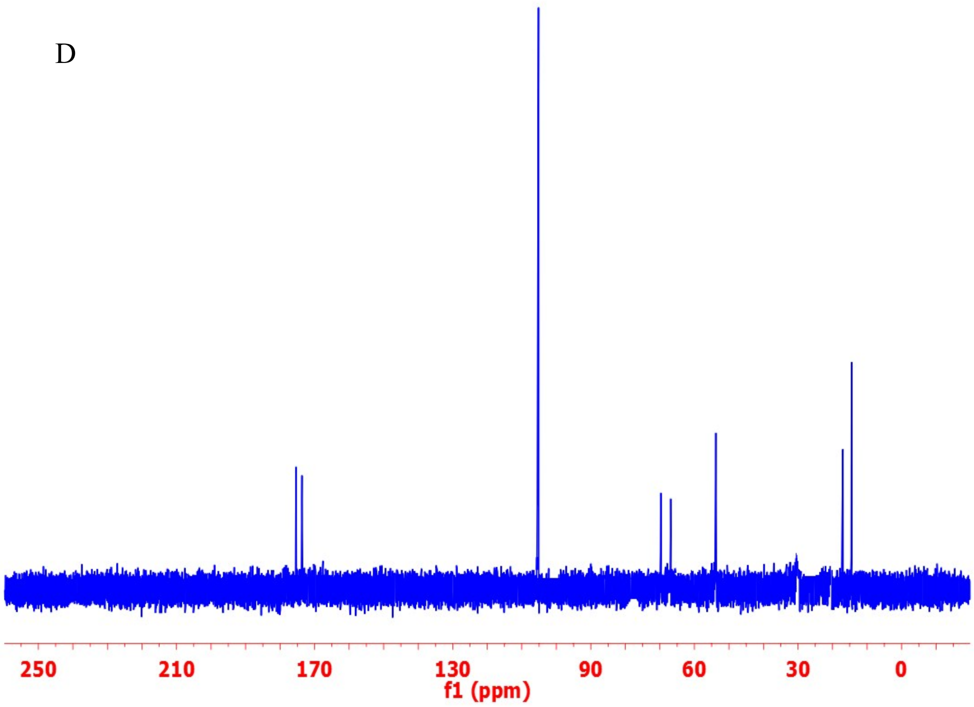

S. Figure 6. ^{1}H, ^{13}C spectrum of NADES 1:3

Swelling studies

The swelling character of the PTX and 7-HC loaded nanocarrier in a variety of buffer solutions at pH 2.8, 5.5, and 7.4 was tested, which is shown in Figure 5. The swelling nature of the nanocarrier at pH 2.8 degraded more compared with the other pH solutions such as 5.5 and 7.4. This is because, at a further acidic pH, there is increased bond breakage between the ester and amide bonds present in the nanocarrier. Furthermore, the free of charge amino group of the lysine moiety in the carrier easily protonated in the acidic environment and it forms NH_3^+ (Praphakar R. A et al., 2018; Mailander V et al., 2009).

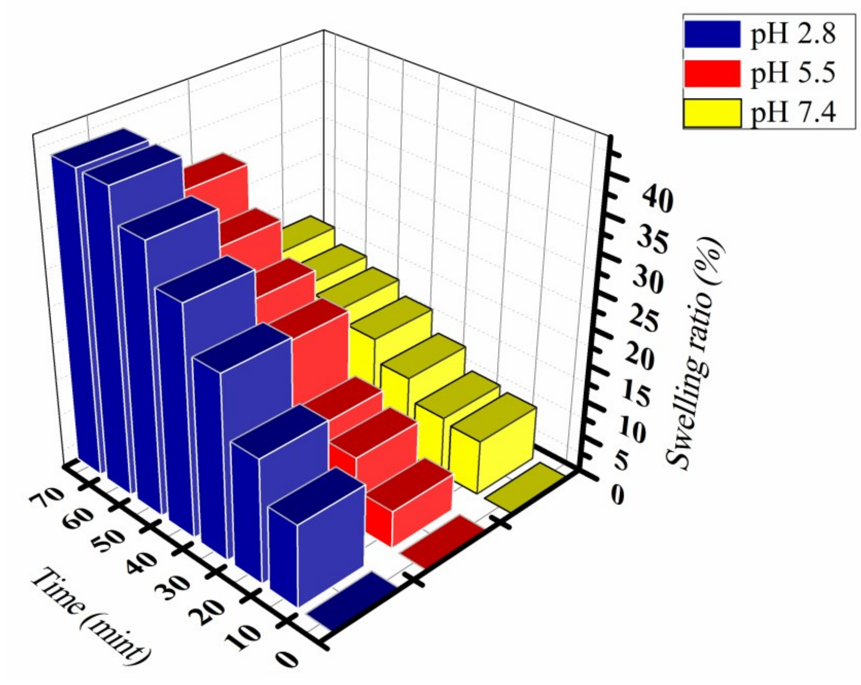

S. Figure 7. Swelling behavior of PTX and 7-HC loaded nanocarriers at various pHs of 2.8, 5.5, and 7.4.

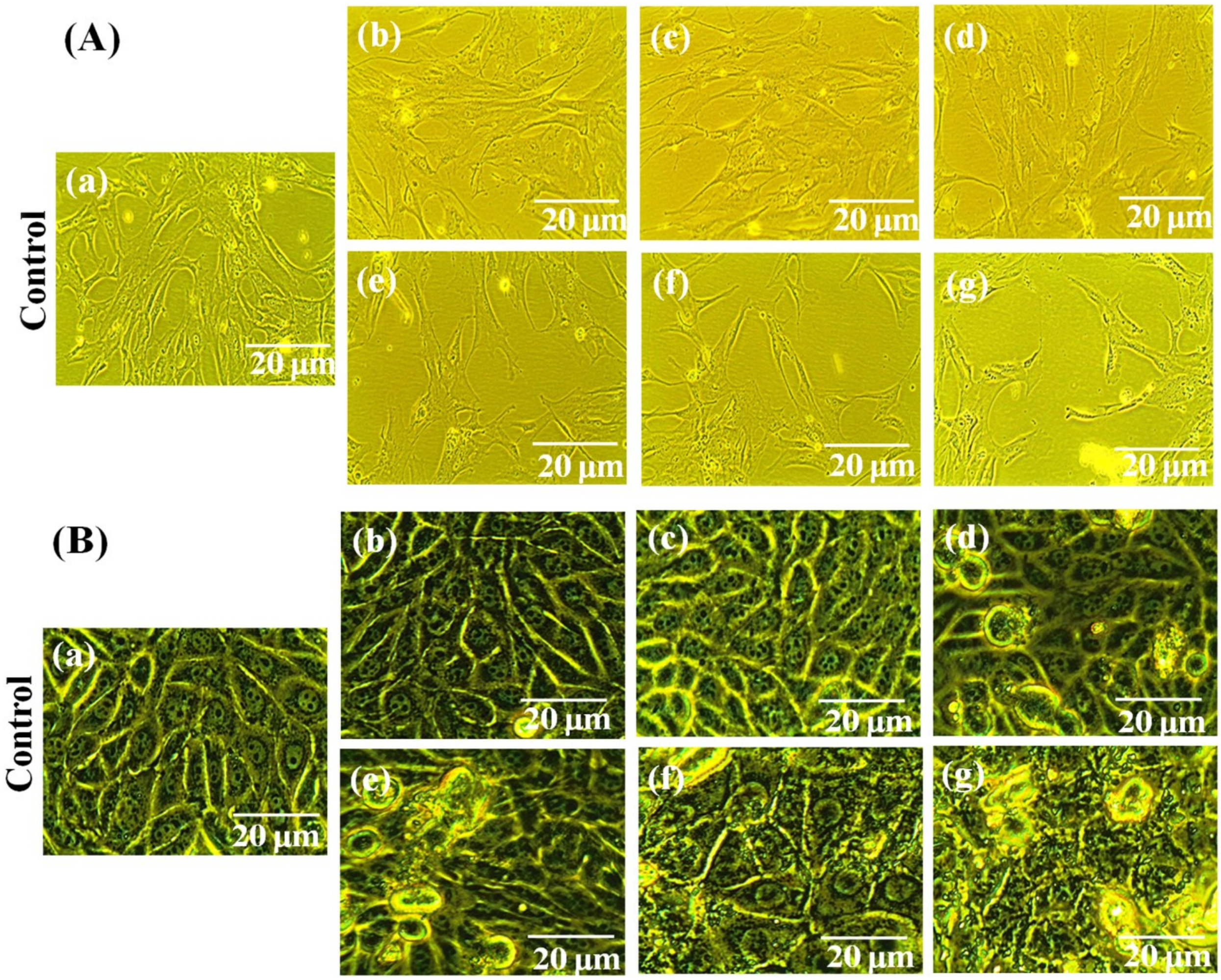

Figure 8. (A) *In-vitro* cell viability studies of PTX and 7-HC loaded SLN in normal cell line- Scale bars = 20 µm. (a) Control, b) SLN, and PTX and 7-HC loaded SLN at various concentrations c) 10, d) 25, e) 50, f) 75, and g) 100 µg/mL^{-1}. (B). *In-vitro* cytotoxicity studies of PTX and 7-HC loaded SLN in MDA-MB-231 cancer cell line- Scale bars = 20 µm. (a) Control, b) SLN carriers, and PTX and 7-HC loaded SLN carriers at various concentrations c) 10, d) 25, e) 50, f) 75, and g) 100 µg/mL^{-1}.

References

1. Chemat F, Anjum H, Shariff A, Kumar P, Murugesan T. (2016) Thermal and Physical Properties of (Choline Chloride + Urea +L-Arginine) Deep Eutectic Solvents. *Journal of Molecular Liquids* 218:301-308.
2. Mailander V, Fester K. L. (2009) Interaction of Nanoparticles with Cells. *Biomacromolecules* 10:2379-2400.
3. Praphakar R. A, Jeyaraj M, Mehnath S, Higuchi A, Ponnamma D, Sadasivuni K. K, Rajan M. (2018) pH-sensitive Guar Gum Grafted Lysine-β-Cyclodextrin Drug Carrier for Controlled Releases on Cancer Cells. J. *Mater. Chem. B.* DOI: 10.1039/C7TB02551C.
